# Episomal HBV persistence within transcribed host nuclear chromatin compartments involves HBx

**DOI:** 10.1186/s13072-018-0204-2

**Published:** 2018-06-22

**Authors:** Kai O. Hensel, Franziska Cantner, Felix Bangert, Stefan Wirth, Jan Postberg

**Affiliations:** 1 Department of Pediatrics, HELIOS University Hospital Wuppertal, Centre for Clinical and Translational Research (CCTR), Faculty of Health, Centre for Biomedical Education and Research (ZBAF), Witten/Herdecke University, Heusnerstr. 40, 42283 Wuppertal, Germany; 2 Department of Paediatric Gastroenterology, Hepatology and Nutrition, University of Cambridge, Addenbrooke’s Hospital, Hills Road, Cambridge,, CB2 0QQ UK; 3 Clinical Molecular Genetics and Epigenetics, Faculty of Health, School of Medicine, Centre for Biomedical Education and Research (ZBAF), Witten/Herdecke University, Alfred-Herrhausen-Str. 50, 58448 Witten, Germany

**Keywords:** Epigenome, Episome, Host–pathogen interaction, Supranucleosomal structure, X-protein, HBxAg, Oncogene, Transcription factories, TADs, Chromatin fiber loops

## Abstract

**Background:**

In hepatocyte nuclei, hepatitis B virus (HBV) genomes occur episomally as covalently closed circular DNA (cccDNA). The HBV X protein (HBx) is required to initiate and maintain HBV replication. The functional nuclear localization of cccDNA and HBx remains unexplored.

**Results:**

To identify virus–host genome interactions and the underlying nuclear landscape for the first time, we combined circular chromosome conformation capture (4C) with RNA-seq and ChIP-seq. Moreover, we studied HBx-binding to HBV episomes. In HBV-positive HepaRG hepatocytes, we observed preferential association of HBV episomes and HBx with actively transcribed nuclear domains on the host genome correlating in size with constrained topological units of chromatin. Interestingly, HBx alone occupied transcribed chromatin domains. Silencing of native HBx caused reduced episomal HBV stability.

**Conclusions:**

As part of the HBV episome, HBx might stabilize HBV episomal nuclear localization. Our observations may contribute to the understanding of long-term episomal stability and the facilitation of viral persistence. The exact mechanism by which HBx contributes to HBV nuclear persistence warrants further investigations.

**Electronic supplementary material:**

The online version of this article (10.1186/s13072-018-0204-2) contains supplementary material, which is available to authorized users.

## Background

Worldwide, hepatitis B virus (HBV) infection is the most common chronic virus infection. Currently, at least 240 million people are chronically infected [1] and approximately 30% of the world’s population show serologic evidence of recent or past infection [2]. With more than 780,000 annual cases, HBV infection is the tenth leading cause of death due to cirrhosis or hepatocellular carcinoma (HCC) [3]. In high prevalence areas, chronic HBV infection is estimated to account for more than 80% of HCC cases [4]. While HBV itself is not cytotoxic, liver damage and viral clearance are rather determined by host immune response and virus replication events [5].

Human HBV belongs to the hepadnaviridae family. Its virus particles contain a partially double-stranded, relaxed circular DNA genome (RC DNA), and HBV features a complex replication cycle: Upon infection mediated by the NTCP receptor, the HBV nucleocapsids become transported to the hepatocellular nucleus, wherein the RC DNA becomes converted into a circular episome, the covalently closed circular DNA (cccDNA). The cccDNA serves as a template for all HBV RNA synthesis and persists in the hepatocyte nucleus [6]. For virus particle production, this double-stranded genome is transcribed as a long pregenomic RNA (pgRNA) and subsequently converted to DNA through reverse transcription. Importantly, current antiviral therapy is not curative as it predominantly targets cytoplasmic reverse transcription of the pgRNA, but not the episomal persistence of cccDNA in the cell nucleus. The latter is root for all progeny virus production and therefore widely seen as a potential target for eradicative therapy concepts [7]. At present, HBV replication can be impaired using type 1 interferon and nucleoside/nucleotide analogues, whereas viral clearance—and thus ultimately cure—would require complete cccDNA decay. Unfortunately, achievement of this goal has been widely hampered by a limited understanding of the molecular mechanisms involved in the nuclear persistence of cccDNA, its functional subnuclear localization and chromatin dynamics involved in its transcriptional activation [8].

Importantly, the sole presence of nuclear cccDNA is not sufficient to establish HBV replication. Instead, the presence of the trans-activating protein HBV X (HBx) is essential to initiate and maintain viral replication after infection. Specifically, nuclear (versus cytoplasmatic) localization of HBx was demonstrated to be sufficient to restore HBx-deficient HBV replication. Moreover, discriminative expression of either nuclear export signal (NES)-HBx or nuclear localization signal (NLS)-HBx was shown to provide a feasible technical assay to study the subcellular functions of cytoplasmic and nuclear HBx [9]. In addition, HBx promotes viral transcriptional activity in a self-perpetuating feedback loop [10], whereas hepatocyte inoculation with HBx-deficient HBV particles results in a lack of productive HBV infection. Strikingly, trans-complementation of HBx leads to a rescued phenotype of hepatocytes infected with HBx-deficient HBV particles—a phenomenon reminiscent of a natural HBV infection with respect to viral transcription as well as antigen and virion secretion [11]. HBx has been further shown to induce several epigenetic modifications of nuclear cccDNA such as nucleosome positioning, DNA methylation and other histone post-translational modifications (PTMs) [12, 13] (Additional file 1: Figure S1). Furthermore, numerous studies have shown that HBx can affect host cell metabolism, and it is seen as a weak oncogene that contributes to hepatocarcinogenesis [14–16]. However, the exact mechanisms underlying HBx-facilitated cccDNA establishment in the hepatocyte nucleus, transcriptional regulation and HCC development remain incompletely understood.

The nucleus is the major taxon-specifying organelle in eukaryotic cells, wherein key processes of viral infections take place. Importantly, the cell nucleus is much more than a bare container of genetic material. It comprises subnuclear architectural features that include approx. 10,000 chromatin fiber loops with a median length of ~ 185 kb (range 40 kb–3 Mb). Clusters of these loops are believed to constitute fundamental topological and functional units on a supranucleosomal level of chromatin organization [17, 18]. CpG signaling and histone modifications contribute to higher-order chromatin structure formation in a region specific fashion, i.e., on individual chromatin fiber loops or topologically associating domains (TADs), respectively. The activity of genes clustered on a given loop is believed to be jointly regulated by a common chromatin structure [18–22]. Multiple TADs can form functional nuclear compartments, depending on their interactions with involved functionally diverse nuclear bodies. Furthermore, nuclear compartmentalization is a result of the separation of condensed chromatin structures from a chromatin-poor interchromatin compartment or network. Hereby, a nuclear landscape is shaped that appears sponge-like and features an extended surface, occasionally referred to as the perichromatin region [23]. This region is believed to be a functional compartment, where actively transcribed chromatin loops become exposed and get in the reach of the molecular machineries involved in essential nuclear processes, such as during gene transcription and RNA processing [19, 23, 24]. Functional spatiotemporal nuclear topology consequently entails a higher level of structural order of the epigenome [25]. It is this four-dimensional nuclear landscape that sets the scene for the dynamic short- and long-range interplay of genomic loci and, importantly, genome invaders such as DNA viruses in general, and HBV in particular. For the big picture, interactions between both viral and host genomes must be interpreted in the context of the entirety and complexity of both epigenomes. Due to the overwhelming complexity of already known epigenetic modifications as well as to their partially transient and often dynamic nature, most studies have focused on specific epigenetic changes of the HBV genome [26–29]. However, the interplay of HBV and the higher-order nuclear architecture of the host cell nucleus remains almost unexplored to date.

This is the first study utilizing circular chromosomal confirmation capture (4C) to investigate the nuclear localization of cccDNA in hepatocytes and its interactions with the host genome and epigenome. Specifically, we performed a proximity-based ligation assay in combination with mRNA-seq and ChIP-seq. Interestingly, the probability of cccDNA presence was found to correlate (on a resolution of approx. 50 kb) with regions of active mRNA transcription and enrichment of post-translational histone modifications that are associated with open chromatin, as well as with nuclear HBx. It thus appears that both the cccDNA and HBx are preferentially associated with structural chromatin units on a supranucleosomal scale, if these occur in a transcriptionally active state. This would fit well within the range of chromatin clusters or TADs organizing an interchromatin compartment [17, 18, 24]. Importantly, ectopically expressed HBx alone associates with open chromatin in the host nucleus, and the HBx coverage exhibits genome-wide overlap with expressed genes and with regions, where Pol2 and active histone marks are enriched. In vitro results indicate that nuclear HBx can bind plasmid-encoded HBV episomes via its C-terminus suggesting that HBx could be involved in the nuclear localization of HBV episomes.

## Methods

### Cell lines, virus particle production and HBV infection

HBV particles were concentrated from the supernatant of HepG2.2.15 cells (own collection, originally kindly provided by Ulrike Protzer Lab, Technical University Munich [TUM], Germany) cultivated in 1720 cm^2^ hyperflasks in Williams E medium with 5% fetal calf serum and 1% DMSO utilizing centrifugal filter devices (Centricon Plus-70, Biomax 100.000, Millipore Corp., Bedford, MA) [30]. HepaRG is a human liver cell line, susceptible to HBV infection, that upon differentiation features characteristics of primary human hepatocytes and thus represents a valuable alternative to ex vivo cultivated primary human hepatocytes for the study of HBV infection [31]. HepaRG cell culture (routinely purchased on demand, Biopredic International, Saint-Grégoire, France), differentiation in the presence of DMSO and HBV infection have been performed as previously described [32, 33]. 5 days post-seeding, HepaRG cells were infected with indicated amounts of HBV particles obtained from HBV-replicating HepG2.2.15 in the presence of 5% polyethylene glycol (PEG) 8000. Frozen virus stocks with an MOI of 400 were used and incubated overnight. Subsequently, HepaRG cells were washed three times to remove remnants of the inoculum. For daily monitoring of HBV replication hepatitis B surface antigen (HBsAg) concentrations in cell culture supernatants were quantified using the Elecsys HBsAg II ELISA test (Roche Diagnostics, Rotkreuz, Risch, Switzerland). Alternatively, HBV genomic DNA was analyzed by PCR using different primer combinations. As the HBV genome can occur either as cccDNA or relaxed circular DNA, primers have been selected in two different fashions that feature either cccDNA or rcDNA sensitivity. Depending on the primer pair selection, PCR gave rise to an approx. 590 bp amplicon, weakly favoring cccDNA by single-strand DNA targeting of one primer (HBV_D3fw: 5′-ctgtaccaaaccttcggacg-3′/HBV_D3rv(ss)1: 5′-gcaacggggtaaaggttcag-3′). Alternatively, an approx. 140 bp amplicon, not favoring cccDNA, was detected (HBV_D3fw: 5′-ctgtaccaaaccttcggacg-3′/HBV_D3rv2: 5′-aaccactgaacaaatggcac-3′) (Additional file 1: Figure S2A). For most experiments, HepaRG cells were harvested 12 days post-infection and used for experiments. Murine non-transformed hepatocytes MMH-D3 as well as human hepatoma cell lines HepG2.2.15 and HepG2H1.3 cells (own collection, originally kindly provided by Ulrike Protzer Lab, TUM, Germany) were cultivated as described elsewhere [34, 35].

### HBV DNA-fluorescence in situ hybridization (FISH)

Probes for HBV DNA-FISH were 3′-labelled with digoxigenin (mixture of 2 probes, 50 ng/µL each: 1. 5′-gttcacggtggtctccatgcaacgt-3′; 2. 5′-aggtgaagcgaagtgcacacggacc-3′). Prior to hybridization, probes and a 50-fold excess of Cot-1 human competitor DNA (Roche) were precipitated with ethanol, air-dried and subsequently dissolved in hybridization mixture (50% formamide, 10% dextran sulfate, 2xSSC). HepaRG cells were grown, differentiated and infected as described above. To perform FISH, cells were detached with trypsin and seeded on coverslips, and allowed to attach for several hours. Cells were then permeabilized with 0.5% Triton X-100-phosphate buffered saline (PBS) for 20 min, followed by incubation with 0.1 M HCl for 5 min at room temperature. Preparations were then equilibrated in 50% formamide in 2× SSC for 7 days at 4 °C. For DNA denaturation, samples were treated with 70% formamide in 2× SSC pH 7.4 for 3 min at 72 °C. Nuclei were then briefly washed in ice-cold 70% ethanol followed by 100% ethanol and finally in 2× SSC for 5 min. The probe was denatured simultaneously in a boiling water bath for 10 min, briefly chilled on ice and subsequently loaded onto a coverslip with immobilized and fixed cells. Hybridization was performed overnight at 42 °C followed by post-hybridization washes in 2× SSC at 42 °C and 0.1× SSC at 64 °C. Blocking was done in 3% bovine serum albumin (BSA), 0.1% Triton X-100, PBS (blocking solution) for 20 min at room temperature. For detection, rabbit anti-DIG-Cy3 polyclonal antibody (Jackson ImmunoResearch) was diluted in blocking solution. Incubation was performed for 1.5 h at 37 °C. To-Pro-3 at 5 µM in PBS was used for DNA counterstaining. Thereafter, Vectashield antifade mounting medium (Vectorlabs) was applied.

### Confocal laser scanning microscopy (CLSM)

Results of GST-HBx immunofluorescence staining and HBV DNA-FISH were analyzed by CLSM. Acquisition of serial sections was done with a Zeiss LSM 5 Pascal confocal laser scanning microscope equipped with water objective lenses (Plan-Neofluar 25/0.8, or in some cases C-Apochromat 63/1.2). Fluorochromes were visualized with an argon laser with excitation wavelengths of 488 nm for Alexa Fluor 488 and 543 nm for Cy3. Fluorochrome images were scanned sequentially generating 8-bit grayscale images. Image resolution was 512 × 512 pixels with variable pixel size depending on the selected zoom factor. The axial distance between light optical serial sections was 300 nm. To obtain an improved signal-to-noise ratio, each section image was averaged from four successive scans. The 8-bit grayscale single-channel images were overlaid to an RGB image assigning a false color to each channel and then assembled into tables using open source software ImageJ (Rasband, W.S., ImageJ, National Institutes of Health, Bethesda, MD, USA, http://rsb.info.nih.gov/ij/, 1997–2004.) and Adobe Photoshop CS5 software.

### Expression of HBx-constructs

To study the nuclear localization and HBV episome-binding properties of nuclear HBx, constructs with an N-terminal nuclear localization signal (NLS) were synthesized (Genscript, Piscataway, NJ, USA) and subsequently cloned into the pTagRFP-N vector (Evrogen, Moscow, Russia), which allowed the expression of HBx fused to a C-terminal RFP-tag. To study the nuclear localization of HBx, pTag-NLS-HBx(D3-wt)-RFP was transfected into HBV-naïve MMH-D3 murine hepatocytes using Lipofectamine 2000 (Life Technologies, Carlsbad, CA, USA), followed by G418 selection for 14 days. The presence of the vector and the expression of the transgene mRNA (via cDNA after reverse transcription) was verified by PCR and agarose gel electrophoresis. After G418 selection, proliferating cells were harvested at approx. 50–70% confluence and used for ChIP experiments. Alternatively, to study the association of HBx and cccDNA, pTag-RFP plasmids containing NLS-HBx(D3-wt) or NLS-HBx(D3-del)-deletion constructs were transfected into HBV-Met murine hepatocytes (own collection, originally kindly provided by Marco Tripodi Lab, Sapienza Università di Roma, Italy) using Lipofectamine 2000 according to manufacturer’s recommendations. Approx. 48 h post-transfection, chromatin was prepared from these cells and used for the co-IP of NLS-HBx-RFP utilizing an RFP-trap coupled to magnetic beads (ChromoTek, Martinsried, Germany).

### RNA purification, transcriptome analyses and focused gene expression arrays

Prior to gene expression analyses, total RNA was isolated from at least three biological replicates for each experiment using Trizol reagent (Sigma Aldrich, St. Louis, MO, USA) according to the manufacturer’s recommendations. RNA integrity was validated using an Agilent Bioanalyzer 2100 with the Agilent RNA 6000 Nano Kit for microcapillary electrophoresis (Agilent Technologies, Santa Clara, CA, USA). We determined RNA transcription profiles of several hepatocyte cell populations (HepaRG, HepG2.2.15, HepG2 H1.3). For mRNA enrichment, we used the NEBNext Poly(A) mRNA Magnetic Isolation Module (NEB) and then the NEBNext Ultra RNA Kit (NEB) for Illumina-compatible library preparation upon manufacturer’s recommendations. For focused expression analyses of selected genes relevant for HCC development, we used the RT2 First Strand kit (Qiagen/SABiosciences, Hilden, Germany) for cDNA template synthesis from 1 μg RNA. Gene expression was analyzed from these cDNAs using RT2 Profiler PCR arrays (PAMM-133R; Qiagen/SABiosciences, Hilden, Germany) containing validated primers for 84 genes relevant for liver cancer (HCC) development (Additional file 1: Table S1), 5 housekeeping genes and quality control primers for estimation of reverse transcription efficiency and genomic DNA contamination on a Corbett Rotor-Gene 6000 qPCR device (Qiagen, Hilden, Germany). The housekeeping genes ACTB, GAPDH and RPL19 were used for normalization. For relative comparative quantification of gene expression fold changes, we applied the ΔΔCt method [36] using at least three housekeeping genes for normalization.

### Chromatin immunoprecipitation and co-immunoprecipitation of HBx-RFP

Briefly, lysates from cells cross-linked for 10 min in 1% formaldehyde were layered onto a 1.6 M sucrose cushion (1.6 M sucrose in 60 mM, KCl 15 mM NaCl, 5 mM MgCl_2_, 0.1 mM ethylene glycol tetraacetic acid (EGTA), 15 mM Tris–HCl (pH 7.5), 0.5 mM DTT, 0.1 mM phenylmethanesulfonyl fluoride (PMSF), 3.6 ng/mL aprotinin) and centrifuged as described elsewhere [37]. Nuclei were collected and portions corresponding to 50 μg chromatin were sheared by ultrasonic treatment using a Bioruptor UCD-200 (Diagenode) and 30 × [30 s ON/30 s OFF] at position ‘high.’ Chromatin fragment size was controlled on agarose gels, and one of the chromatin aliquots was saved as input. For ChIP analyses, sheared chromatin samples were incubated with the respective antibody-of-interest in a rotator for 16 h at 4 °C. Subsequently, 20 μL protein G magnetic beads (Diagenode) were added and incubated for 4 h rotating at 4 °C. Immunocomplexes attached to protein G magnetic beads were separated using a magnetic rack and washed repeatedly. To elute DNA fragments enriched by immunoprecipitation, immunocomplexes were incubated with elution buffer [1% SDS, 10 mM EDTA, 50 mM Tris–HCl, (pH 8.1)] for 30 min at 65 °C on a shaker. Eluted immunocomplexes were treated with proteinase K overnight at 55 °C. Chromatin isolation and ChIP analyses were carried out as previously described [38]. Antibodies used for IP were directed against HBxAg (mouse monoclonal antibody, clone 3F6-G10; Bio-Rad Laboratories Inc.) [39], Pol2 (Active Motif, Carlsbad, CA, USA), H3K4me3 (Diagenode, Liège, Belgium), H3K4me1 (Diagenode, Liège, Belgium), H3K9me3 (Diagenode, Liège, Belgium), H3K9ac (Diagenode, Liège, Belgium), H3K27me3 (Diagenode, Liège, Belgium), H3K27ac (Diagenode, Liège, Belgium), H3K36me3 (Diagenode, Liège, Belgium) or H3K36ac (Diagenode, Liège, Belgium). DNA associated with enriched immunocomplexes was purified by phenol:chloroform:isoamylic alcohol extraction and precipitated with isopropylic alcohol. Thereafter, Illumina-compatible libraries were prepared using the MicroPlex Library Preparation kit v2 (Diagenode, Liège, Belgium). Massive parallel sequencing was performed as described below. In similar ways nuclei and chromatin were treated for co-IP experiments to investigate whether the diverse NLS-HBx-RFP-constructs (wt and deletion constructs) were associated with HBV episomes. An RFP-trap coupled to magnetic beads (ChromoTek, Martinsried, Germany) was used for enrichment of NLS-HBx-RFP complexes. DNA associated with enriched complexes was purified by phenol:chloroform:isoamylic alcohol extraction and precipitated with isopropylic alcohol. Thereafter, the relative enrichment of co-precipitated episomal HBV DNA sequences was assessed using qPCR (HBV_cccDNAfw(579): 5′-gactctctcgtccccttctc-3′/HBV_cccDNArv(579): 5′-atggtgaggtgaacaatgct-3′, or HBV RC/cccDNAfw(100): 5′-gttgcccgtttgtcctctaattc-3′/HBV_RC/cccDNArv(100): 5′-ggagggatacatagaggttccttga-3′, respectively) and normalized to the amount of host genomic DNA (Primer mix ‘human LMNA promoter’ (Diagenode, Liège, Belgium, cat. #pp-1011) and transfected plasmids (RFPfw(92): 5′-tcaaggaggccgacaaagag-3′/RFPrv(92): 5′-aagtttgtgccccagtttgc-3′).

### Monitoring HBx/HBV-reporter plasmid DNA interactions using F2H reporter assays

The coding sequences of NLS-HBx-RFP-constructs (wt and Δ*D*) were cloned into pTOPO-GFP plasmids (Invitrogen), which enabled their expression as tagged proteins fused with GFP. F2H hamster cells (Chromotek, Martinsried, Germany) provided an in vitro protein–protein interaction assay system carried out in living mammalian cells, which we modified to study the interaction of HBx with reporter plasmid DNA containing the 1.3-fold HBV genome sequence (pCH-9/3091) [40]. F2H cells were co-transfected with plasmids expressing the HBx-GFP construct of interest in combination with pCH-9/3091 containing a terminally redundant genome of HBV (subtype ayw 1, kindly provided by Ulrike Protzer, TU Munich) and a platform reagent. F2H nuclei constitute a GFP-binding matrix, leading to the immobilization of GFP-fusion proteins at an enforced nuclear localization. Using CLSM we sequentially monitored the nuclear localization of HBx-GFP in living cells and the nuclear localization of pCH-9/3091 by FISH in fixed cells. CLSM and FISH were done as described above. Interactions between HBx-GFP and pCH-9/3091 were visualized as co-localization of green and red fluorescent signals at the nuclear GFP-binding matrix.

### Circular chromosome confirmation capture (4C)

In order to select a restriction strategy applicable for multiple HBV genotypes, we performed alignments using ClustalW included in molecular evolutionary genetics analysis (MEGA) 4.1 [41] (Additional file 1: Figure S2B). The genotype HBV D3, which is replicated by HepG2.2.15 cells and used for the infection of HepaRG cells, was verified by PCR using primers that give rise to an approx. 590 bp amplicon (depending on the HBV genotype) as described above. Subsequently, Sanger sequencing was utilized for amplicons cloned into pGEM-T easy (Promega) via the standard T7 sequencing primer.

4C targeting on HBV cccDNA (verified genotype D3) as bait was conducted as described previously [42, 43] with slight modifications (Additional file 1: Figure S2C). Briefly, interacting DNA segments were cross-linked using 1% formaldehyde and nuclei were isolated using a lysis buffer containing 0.4% Tergitol (NP-40 substitute) followed by incubation at 37 °C in the presence of 0.3% SDS. Prior to the primary digestion step SDS was sequestered using 2% Triton X-100, and subsequently, SpeI (NEB, 600U, 24 h) was added. Digestion efficiencies were quantified by qPCR using a Corbett Rotor-Gene 6000 qPCR device and Quantitect SYBR Green Master Mix (Qiagen, Hilden, Germany) with primers covering the SpeI restriction site within the cccDNA normalized to a non-digested region. Digested samples were purified, diluted and then proximity-ligated at 16 °C using T4 DNA ligase (Roche). For 4C library construction the crosslink was reversed. Subsequently, samples were subjected to digestion with HaeIII (NEB, 20 U per µL, 24 h) followed by a second ligation. Using specific primer pairs (Primer A: 5′-cccactgtttggctttcag-3′/Primer B: 5′-gggaaagccctacgaaccac-3′, or, respectively, Primer C: 5′-gtttctcctggctcagtttac-3′/Primer D: 5′-gtttctcytggctcagtttac-3′) adjacent to the respective ligation sites, genomic sequences associated with HBV cccDNA were amplified. From these amplicons Illumina-compatible libraries were made using the MicroPlex Library Preparation kit v2 (Diagenode, Liège, Belgium). Massive parallel sequencing was performed as described below.

### Massive parallel sequencing, data handling and bioinformatics

Analyses of 4C libraries were performed using the paired-end read option (42 nt) and the Illumina NextSeq 500 High Output Kit. Analyses of transcriptomes and ChIP-seq were conducted with the single read option (75 nt) and the Illumina NextSeq 500 High Output Kit. For processing of all raw data, the following data analysis pipeline was selected: 1. bcl2fastq2 v2.15.0 for demultiplexing; 2. Fastqc 0.10.1 for read quality assessment; 3. Cutadapt-1.2.1 for adaptor trimming. This work has benefited from the facilities and expertise of the high-throughput sequencing core facility of Imagif (Research Center of Gif—www.imagif.cnrs.fr).

Fastq files were uploaded onto the Galaxy server and converted using FASTQ Groomer [44] prior to read mapping (*Mus musculus* genome mm9 or *Homo sapiens* hg19, respectively) using Bowtie2 with the ‘very sensitive end-to-end’ option. The resulting BAM file was used for direct visualization of mapped reads in Geneious R9 software or further processed for comparative analyses using a deepTools [45] pipeline (1. bamCoverage [variable bin sizes: 2, 10, 50 and 250 kb] to generate coverage bigWig files for visualization in the UCSC genome browser (genome.ucsc.edu) or, alternatively, bedgraph tabular files for correlation analyses; 2. bigwigCompare with default options to normalize and compare two bigwig files and visualize the results in the UCSC genome browser. Alternative color grading look-up tables (LUT) contained in WCIF ImageJ software were assigned to the UCSC genome browser grayscale dense coverage tracks for improved visualization.

### HBx-mRNA silencing

For HBx-mRNA silencing 2′-deoxy-2′-fluoro-beta-d-arabinonucleic acid (FANA) antisense oligonucleotides (AUM BioTech, Philadelphia, PA, USA) were used [46]. Target sequences were chosen to match exclusively a stretch of the HBx encoding sequence not overlapping with other HBV genes (Hepatitis B virus; genotype D3 [FANA target sequences underlined]: cgcccaccaaatattgcccaaggtcttacataagaggactcttggactctcagcaatgtcaacgaccgaccttgaggcatacttcaaagactgtttgtttaaagactgggaggagttgggggaggagattaggttaaaggtctttgtactaggaggctgtaggcataaattggtctgcgcaccagcacc). As scrambled control FANA, respectively, TGACCCTATGCTGTTCCTATA was selected. Briefly, FANA antisense oligonucleotides were delivered to each 2.5 *10exp5 HepG2.2.15 cells per well by gymnotic delivery upon manufacturers recommendations (500 nM, 2.5, 5 µM). Sampling was done at several time points post-FANA delivery, i.e., 0, 24, 48, 72 h. DNA was isolated using the QIAamp DNA Mini Kit (Qiagen, Hilden, Germany). Subsequently, cccDNA was analyzed by qPCR as described above using the HBV_cccDNAfw(579)/HBV_cccDNArv(579) primers and normalized using a human genomic amplicon (79 bp) from the NOS3 gene promoter (primers: eNOS_TSS(79 bp) + ; 5′-cacaagactccagggaagca-3′/eNOS_TSS(79 bp)-; 5′-ctgcagaaggtgctggtgg-3′). Notably, while we monitored very similar results with the differently applied FANA concentrations, we observed a slight increase in dead cells at 2.5 and 5 µM. Our results were therefore calculated based on experiments, in which 500 nM FANAs were applied.

## Results

### Genome-wide coverage of ectopically expressed HBx correlates with active histone modifications and RNA polymerase 2 at transcribed gene loci in non-transformed murine hepatocytes

**Fig. 1:**
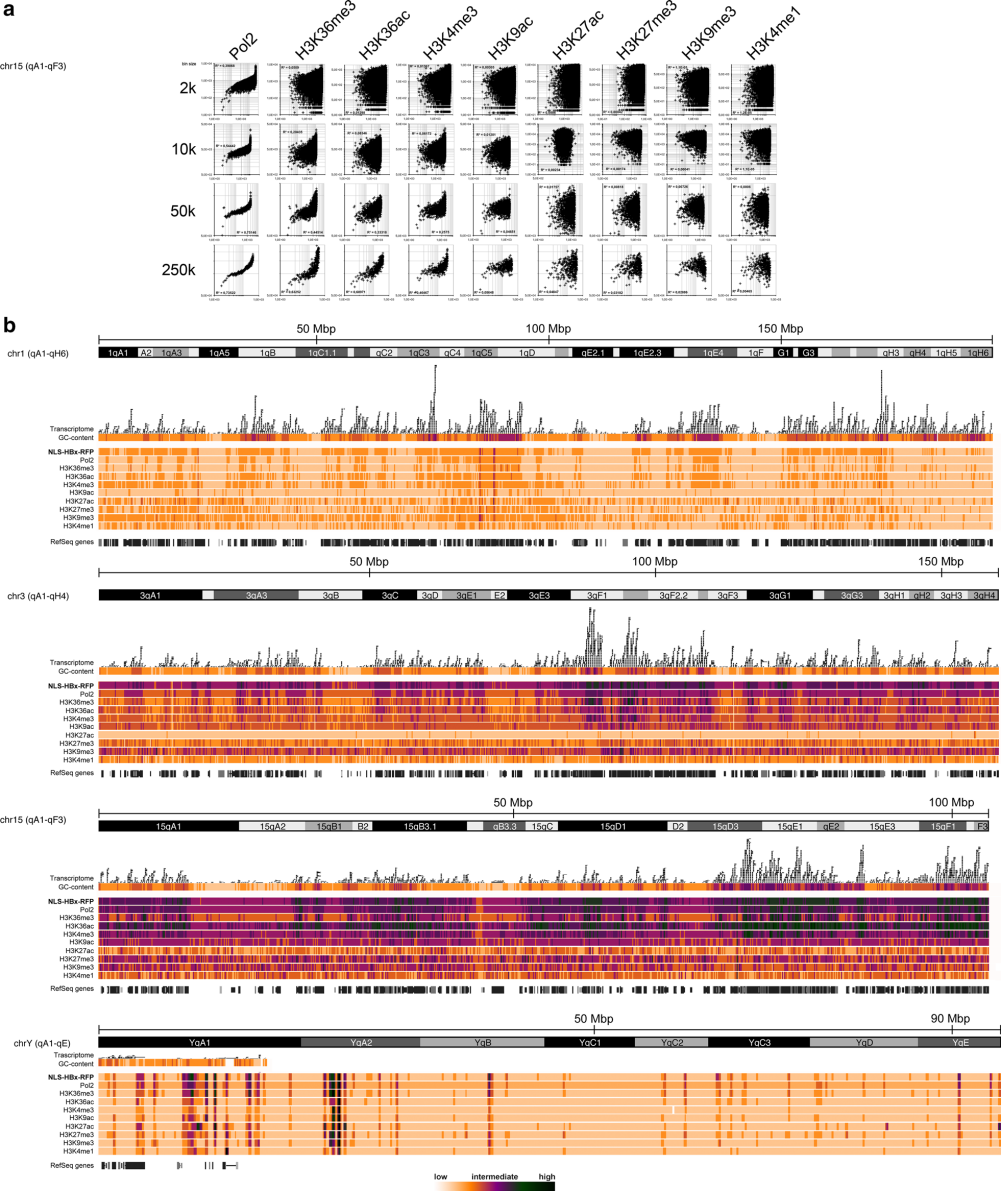
Correlative analyses of HBx-hepatocyte genome association, transcriptomics, Pol2 and several chromatin structure markers on chromosomes 1, 3, 15 and Y in MMH-D3 cells. **a** We performed correlation analyses of NLS-HBx-RFP with respect to Pol2 and several histone modifications. To assess the resolution of putative correlation, we applied different bin sizes for analyses. The graphs illustrate exemplarily for chromosome 15, how R^2^ increases for Pol2, H3K36me3, H3K36ac and H3K4me3, when larger resolution windows of 50 or 250 kb were selected. **b** This sub-figure comprehensively outlines massive parallel sequencing derived whole-genome data for chromosomes 1, 3, 15 and Y (from top to bottom). For each chromosome, the following data are presented (in the order from top to bottom): chromosomal structure, transcriptome, GC-content (both obtained from the UCSC genome browser), ChIP-seq derived enrichment of Pol2, NLS-HBx-RFP, H3K36ac, H3K36me3, H3K4me3, H3K4me1, H3K27ac, H3K27me3, H3K9ac and H3K9me3. Below, the position of RefSeq genes is indicated (obtained from the UCSC genome browser). The degree of enrichment is color-coded as indicated in the legend on the bottom of the figure. On four different chromosomes, this figure demonstrates a correlation using a 50 kb bin size for analyses of HBx mapping and gene transcription, Pol2 mapping as well as enrichment of PTMs that have been associated with active gene transcription. In contrast, repressive PTMs do not exhibit a correlative mapping pattern with HBx enrichment

We hypothesized that HBV genomic DNA and HBx exhibit a specific intra-nuclear localization pattern. This may point to a functionally relevant virus–host interplay in the interphase nucleus, in which the establishment of episomal HBV persistence might depend on HBx and a specific subnuclear localization in similar ways as described for episomal plasmid DNA [47, 48]. To confirm whether HBx exhibits a functionally specific intra-nuclear localization behavior on the molecular level, which may hold for an entire cell population on a genome-wide scale—and not only for a limited number of microscopically inspected cell nuclei—we expressed HBx-RFP in MMH-D3 cells. The nuclear function of HBx was discriminated from the cytoplasmatic function, by an attachment of a nuclear localization signal (NLS) to the N-terminus. We utilized chromatin immunoprecipitation in combination with massive parallel sequencing (ChIP-seq) to study the genome-wide chromatin association of NLS-HBx-RFP. Prior to that, we performed G418-selection of successfully transfected cells. In parallel to NLS-HBx-RFP, nucleosomes containing histones with several post-translational modifications (PTMs) were pulled down using ChIP-seq-validated antibodies. Those antibodies were directed against H3K4me3, H3K4me1, H3K9me3, H3K9ac, H3K27me3, H3K27ac, H3K36me3 or H3K36ac and, furthermore, against RNA polymerase 2 (Pol2). Figure 1 quintessentially illustrates the NLS-HBx-RFP correlation (Fig. 1a) and genomic coverage (Fig. 1b) of all these hallmarks for murine chromosomes 1, 3, 15 and Y. Importantly, the gene-poor chromosome Y—in contrast to the autosomes—exhibits very low signal strength over almost its entire length. To address the question, whether HBx could be associated with specific genes, or alternatively, with higher-order chromatin structures upon the level of specific genes, we performed correlation analyses of NLS-HBx-RFP with different markers at several levels of resolution (i.e., bin sizes of 2, 10, 50 or 250 kb). Interestingly, we observed a clear tendency of positive correlation between NLS-HBx-RFP and Pol2 with best matches within relatively large analysis windows (bins) of 50–250 kb resolution (Fig. 1a). This would fit well to the dimensions observed for higher-order chromatin structures reminiscent of chromatin fiber loops or TADs (40 kb–3 Mb). To determine the underlying chromatin signature, we examined the coverage of multiple histone PTMs characteristic for actively transcribed or repressed genes, respectively. Similarly to Pol2, a positive correlation between NLS-HBx-RFP and ‘active chromatin’ hallmarks, such as H3K36me3, H3K36ac or H3K4me3 was observed. These areas overlapped with GC-rich genomic regions and transcribed areas, which frequently correlated with gene density rather than with extended intergenic sequences (Fig. 1b).

Taken together, we observed a high degree of overlap with local enrichment areas of NLS-HBx-RFP and Pol2 as well as H3K36me3, H3K36ac and H3K4me3 and mRNA synthesis, which matched best to correlation analyses on a supranucleosomal scale (bin sizes 50 kb or 250 kb). In contrast, this correlation became weaker, when smaller bin sizes were selected (Fig. 1a). On the other hand, ChIP-seq analyses of repressive chromatin marks (H3K9me3 and H3K27me3) but also H3K9ac, H3K27ac and H3K4me1 yielded a much more distorted signal at all levels of resolution, poorly corresponding to NLS-HBx-RFP coverage peaks or actively transcribed genes (Fig. 1a, b).

Importantly, our results suggest that NLS-HBx-RFP alone in the absence of cccDNA and other HBV factors was associated with chromatin in the host nucleus. In particular, NLS-HBx-RFP was preferentially enriched in nuclear subdomains, which were associated with several histone PTMs characteristic for open chromatin and Pol2, which ultimately points to sites of active gene transcription on a higher-order level of chromatin organization.

### NLS-HBx-RFP co-precipitates with chromatin and its association with HBV episomes in human hepatoma cells depends on the C-terminus

**Fig. 2:**
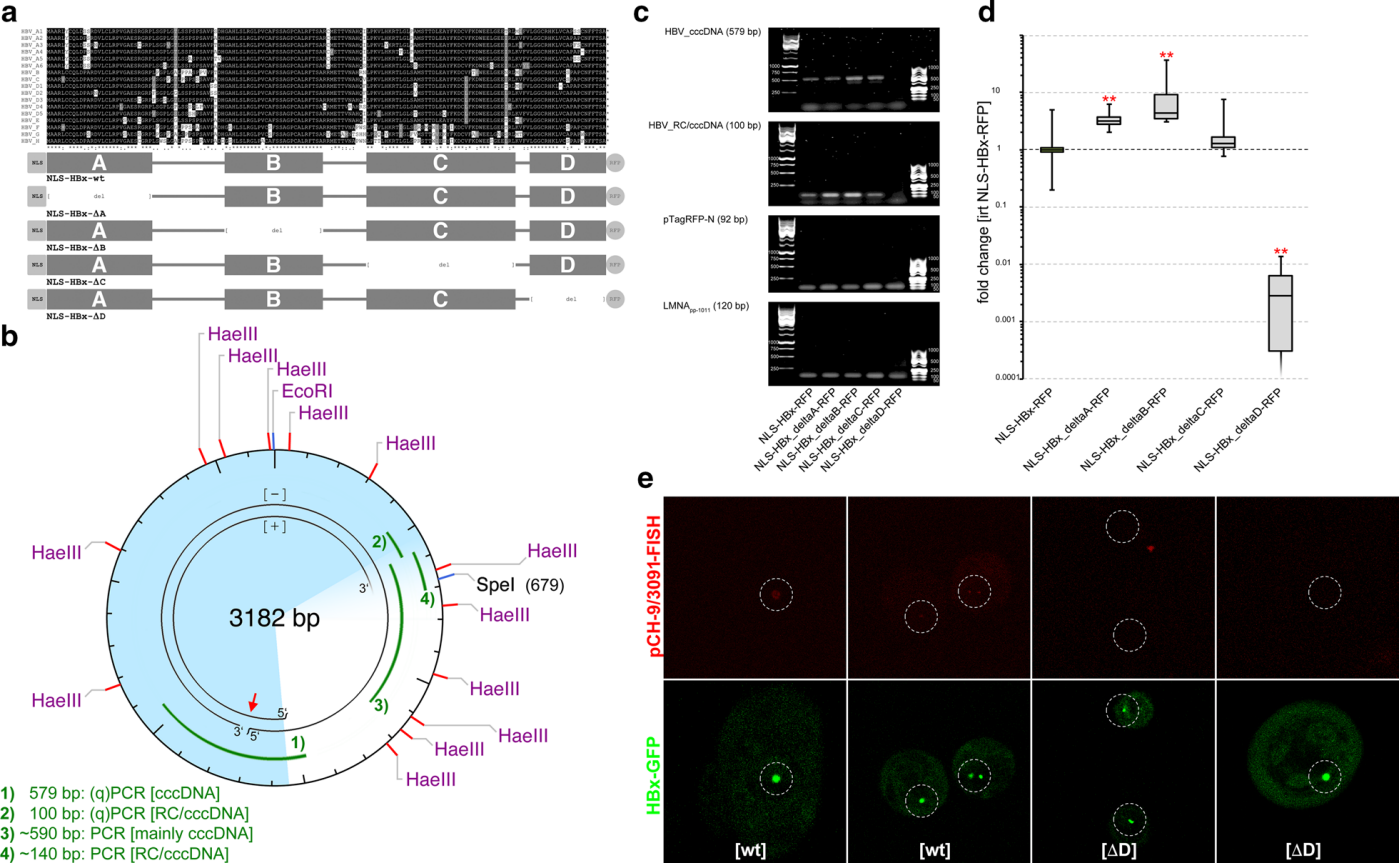
Co-immunoprecipitation of several transfected HBx-constructs and cccDNA as well as co-localization analyses using the F2H cell system. **a** HBV occurs as several different genotypes and sub-genotypes. **a** Shows the alignment of HBx protein sequences for HBV genotypes A–H including sub-genotypes. Sequential agreement is shaded in black, common variations are gray, single differences are highlighted white. This figure demonstrates four regions with a large degree of agreement (indicated as blocks A–D below the protein sequences) and variation mostly between those regions. Outlined below is a scheme of several HBx-constructs that were used in subsequent experiments. Specifically, the constructs feature a nuclear localization signal as well as an RFP signal and either the full-length HBx (first row) or HBx depleted by blocks A, B, C or D (rows 2–5). As a control, full-length HBx-RFP with a nuclear export signal was used (not shown in this figure). **b** This genomic HBV map illustrates the features of RC DNA, the position of restriction sites of interest as well as the amplified regions (1–4; green lines) targeted by specific primer pairs used in this study. The red arrow points to the nick region of the minus-RC DNA strand. **c** Analyses of cccDNA association with nuclear HBx-RFP complexes enriched by immunoprecipitation. Results are shown from semiquantitative PCR HBV cccDNA which was amplified with two different primer pairs resulting in 579 bp (genotype D3) or 100 bp amplicons, respectively. RFP (92 bp) amplicons were used to assess the plasmid load; Lamin A/C (LMNA, 120 bp) amplicons were used to assess the amount of host genomic DNA per sample. Notably, the signal for cccDNA when HBx-Δ*D*-GFP was immunoprecipitated is significantly weaker when compared to the other, clearly visible bands. **d** qPCR derived analysis of cccDNA enrichment utilizing IP directed against HBx-RFP. This figure outlines the fold change in gene expression of cccDNA, normalized to the cccDNA signal when full-length HBx-RFP was pulled down. Importantly, the signal for HBx-Δ*D*-GFP derived cccDNA is significantly attenuated. **Indicates *p *< 0.05. **e** Results of sequential image acquisition in F2H assays, where HBx(wt)-GFP spots co-localized with faint HBV genome FISH signals (left image quartet). In contrast, no HBV genome FISH signals could be identified, when HBx-Δ*D*-GFP was used for co-transfection (right image quartet), although the GFP spots were not distinguishable in both experiments. The phase contrast-like field of view was used to identify overlapping slide areas before and after FISH (not shown). Dashed circles mark areas with nuclei where the GFP-binding platform was localized

To characterize the coherence of the above results and to study the binding of NLS-HBx-RFP to genomic HBV DNA or episomes, respectively, we expressed several NLS-HBx-RFP-constructs in HepG2.2.15 hepatocytes (Fig. 2a). Cells were cultured under defined conditions as described previously to facilitate the production of infectious HBV virions and to optimize the presence of episomal cccDNA, which is usually present in low copy numbers [30, 32, 33]. Deletion constructs of HBx were created on the basis of highly conserved regions determined by amino acid (aa) sequence comparisons between the 154 aa long HBx variant proteins from the major genotypes A-H, including sub-genotypes (Fig. 2a). We recognized four evolutionarily almost invariant target regions (Fig. 2a, blocks A–D) and designed full-length HBx as well as several partial deletion constructs of HBx with an N-terminal nuclear localization signal and C-terminal RFP-tag. As a control, a full-length HBx-RFP preceded by a nuclear export signal (NES-HBx-RFP) was used. Immunofluorescence microscopy was utilized to verify successful transfection and expression of RFP-fusion proteins (Additional file 1: Figure S3).

Subsequently, for the enrichment of cccDNA (over RC DNA), we isolated chromatin from purified hepatocyte nuclei, which were yielded by centrifugation of lysed cells through 1.5 M sucrose cushions [37]. Subsequently we performed immunoprecipitation (IP) using RFP as the antigen followed by PCR and qPCR to evaluate, whether HBV DNA was enriched within the NLS-HBx-RFP immunocomplexes. Therefore, we used primers, which at least partly spanned the nick region present in HBV RC DNA and targeted a region, which occurs as a single strand in RC DNA (Fig. 2b: amplicon 1, 579 bp) [7, 49], or for redundancy, primers not selective for RC or cccDNA (Fig. 2b: amplicon 2, 100 bp). Using these primers for semiquantitative PCR and agarose gel electrophoresis, we observed enrichment of genomic HBV DNA in HBx(wt)-IPs as well as for HBx-ΔA, HBx-ΔB and HBx-ΔC. In contrast, HBx-Δ*D*-IP did not yield detectable amounts of HBV DNA (Fig. 2c). Subsequently, we performed qPCR to quantify HBV DNA enrichment in the precipitated NLS-HBx-RFP immunocomplexes (Fig. 2d). Confirming the results of semiquantitative PCR, immunoprecipitation of HBx lacking the D region yielded a significantly lower amount of associated HBV DNA. HBx-ΔC-IP revealed similar amounts of associated HBV DNA as observed for HBx(wt)-IP. Interestingly, HBx-ΔA-IP and HBx-ΔB-IP appeared to be associated with significantly more HBV DNA. Importantly, since at least amplicon 1 should have derived preferentially from cccDNA, it appeared that NLS-HBx-RFP was associated with the HBV genome in form of cccDNA, and binding involved the HBx C-terminus. It is noteworthy that our experimental approach did not necessarily point to a direct DNA-binding activity of HBx, but rather on its physical linkage with HBV episomes in a chromatin conformation. This is in agreement with previously published data, indicating the association of HBx with cccDNA and nucleosomes containing acetylated histones, histone acetyltransferase p300 and transcription factors. Interestingly, this association was shown to contribute to the formation of an episome adopting a transcriptionally competent chromatin structure [13]. Moreover, we could not predict whether HBx exhibits discriminative binding of specific sequences associated with HBV chromatin over host nuclear DNA, whereas this seemed to be improbable due to the apparent non-sequence-specific association of HBx with host genomic sequences.

To complement the above-described observations, we analyzed the co-localization of HBV DNA and HBx in a different approach. Specifically, we co-transfected HBV DNA encoded on the plasmid pCH-9/3091 [40] with the mammalian expression vector pTag-HBx-GFP2-N (encoding HBx(wt)-GFP or, alternatively, HBx-Δ*D*-GFP) in a mammalian nucleus. The nuclear localization of HBx-GFP was constrained to a dot-like GFP-binding platform in F2H hamster cells (Chromotek). As a first step, we monitored the nuclear localization of HBx-GFP in living cells by immunofluorescence microscopy. Subsequently, to analyze the nuclear localization of a co-transfected pCH-9/3091, we fixed the cells and visualized the HBV genome localization in the same nuclei by FISH (Fig. 2e). Interestingly, FISH signals overlapped well with the prominent dot-like GFP signals in cells expressing full-length HBx(wt)-GFP (Fig. 2e, left image quartet). In contrast, we did not observe HBx-GFP/HBV genome co-localization in cells expressing HBx-Δ*D*-GFP (Fig. 2e, right image quartet). These observations support the results previously obtained by PCR: NLS-HBx(wt)-RFP co-precipitated with the HBV DNA, which at least partly occurred as cccDNA, whereas this interaction was not observed, when the C-terminus was truncated in NLS-HBx-Δ*D*-RFP. Importantly, these results did not point to direct DNA binding of plasmid DNA, which probably became quickly associated with nucleosomes in cycling mammalian cell nuclei. Furthermore, these experiments did not show HBx(wt)-GFP to bind to HBV genomes exclusively.

As a control, we investigated the biological activity of ectopically expressed NLS-HBx-RFP (i.e., whether the different HBx-constructs act as transregulators of host cellular genes reminiscent of HBx in vivo). We analyzed the expression of 84 genes relevant for the development of HCC in stably transfected MMH-D3 hepatocytes by qPCR analyses on cDNA libraries (Additional file 1: Figure S4). Whole RNA was purified and converted to cDNA. RNA from MMH-D3 cells expressing HBx(wt)-RFP with an N-terminal nuclear export signal (NES-HBx(wt)-RFP) was used for normalization to subtract potential indirect effects of cytoplasmic HBx from direct effects of nuclear HBx on the regulation of gene activity. When compared to NES-HBx-RFP, all MMH-D3 cells transfected with NLS-HBx-RFP-constructs exhibited a distinct pattern of up- or downregulated gene activity (to a different extent). These data suggest the diverse HBx-constructs to have retained their biological activity, similarly as reported for HBx previously [50]. Unsupervised hierarchical clustering revealed that the gene expression patterns were most reminiscent of controls (NES-HBx-RFP) when NLS-HBx-Δ*D* was ectopically expressed. This suggests the C-terminus of HBx to be involved both in DNA binding and in the promotion of hepatocellular carcinogenesis, as previously hypothesized by others [51].

### Chromosomal confirmation capture (4C) reveals HBV episome enrichment at host genomic regions of active gene transcription

It is a matter of perspective whether cccDNA is interpreted as a recruiting platform for factors that constitute the persistent HBV episome [13, 52] or, in contrast, whether the architecture of the host nucleus is seen as the compartmentalized, functional chromatin landscape, where the HBV episome needs to assimilate for long-term establishment. The former perspective would suggest the HBV episome-constituting factors to be relatively freely available in the host nucleus. This, however, is not in agreement with current views on a higher-order nuclear architecture. Considering the latter perspective, our observations on the nuclear localization of NLS-HBx-RFP in murine hepatocyte nuclei and its association with HBV episomes in human hepatoma cells allowed us to frame a working hypothesis: An interaction of the cccDNA with HBx in the nucleus might lead to the constitution of an episome, which subsequently becomes assimilated to the transcriptionally active host chromatin landscape. This in turn may result in the physical inclusion into a transcriptionally competent nuclear compartment. Notably, due to HBV’s very small genome size of approx. 3.2 kb it is improbable that the HBV episome is sufficient to adopt a higher-order chromatin structure reminiscent of chromatin fiber loops or TADs. We speculated the HBV episome to be packed into transcriptionally competent chromatin, i.e., follow a hitchhiking principle, where it becomes attached and assimilated to discrete topological units inherent to the host nuclear architecture. Such a mechanism might be important for the persistent establishment of the HBV infection and might also be transiently relevant for the RC DNA before the conversion to cccDNA. This working hypothesis would postulate HBV episomes to exhibit a similar nuclear localization behavior as observed for ectopically expressed NLS-HBx-RFP.

To test this, we utilized genome-wide proximity ligation-based circular chromosomal conformation capture technology in combination with massive parallel sequencing (4C-seq). This approach allowed us to elucidate interactions of HBV episomes (with emphasis on the cccDNA) with the host cellular genome [42, 43]. We analyzed how the small HBV episomes interact with unknown DNA elements on the host cells’ chromosomes. In brief, the 3D chromatin conformation within the hepatocyte nuclei was cross-linked, followed by a 6-base cutter endonuclease digest. The resulting fragments were subsequently ligated, whereby fusion events between bait (here: HBV cccDNA) and prey (hepatocyte chromosomal DNA) occurred, when cccDNA and a given host locus were in close proximity. Subsequently, a second 4-base cutter digest was used for small fragment production. A sub-fraction of these fragments consists of joined bait/prey DNA, which became circularized in a final ligation step. Bait-specific primers were used to amplify libraries of connected prey-sequences for subsequent analyses utilizing massive parallel sequencing (Additional file 1: Figure S2).

Differentiated HepaRG cells were infected with HBV inocula, and the HBV virion-producing hepatoma cell lines HepG2.2.15 and HepG2 H1.3 were used for comparison. RNA-seq derived host cellular gene expression results are displayed in Additional file 2: Table S2. Importantly, both HepG2 cell lines contain HBV sequences integrated into the host genome, rendering them ‘contaminated’ with non-episomal HBV DNA. Furthermore, controversial results about the RC DNA/cccDNA ratio exist for HepG2.2.15 and HepG2 H1.3 cells. Therefore, the results presented here require a careful interpretation, i.e., considering which episomal form of HBV was in the focus of the conducted experiments. For 4C, we selected the unique SpeI restriction site around position 679 of the HBV genome as the best 6-base cutter restriction endonuclease target, because it is flanked by two adjacent HaeIII sites framing a sequence motif optimal for bait primer design (Fig. 2b; Additional file 1: Figure S2B). Moreover, all three restriction sites were selected to correspond with high probability to the single-stranded region of the minus-strand [7, 53], thus selectively favoring the endonuclease digests for cccDNA.

**Fig. 3:**
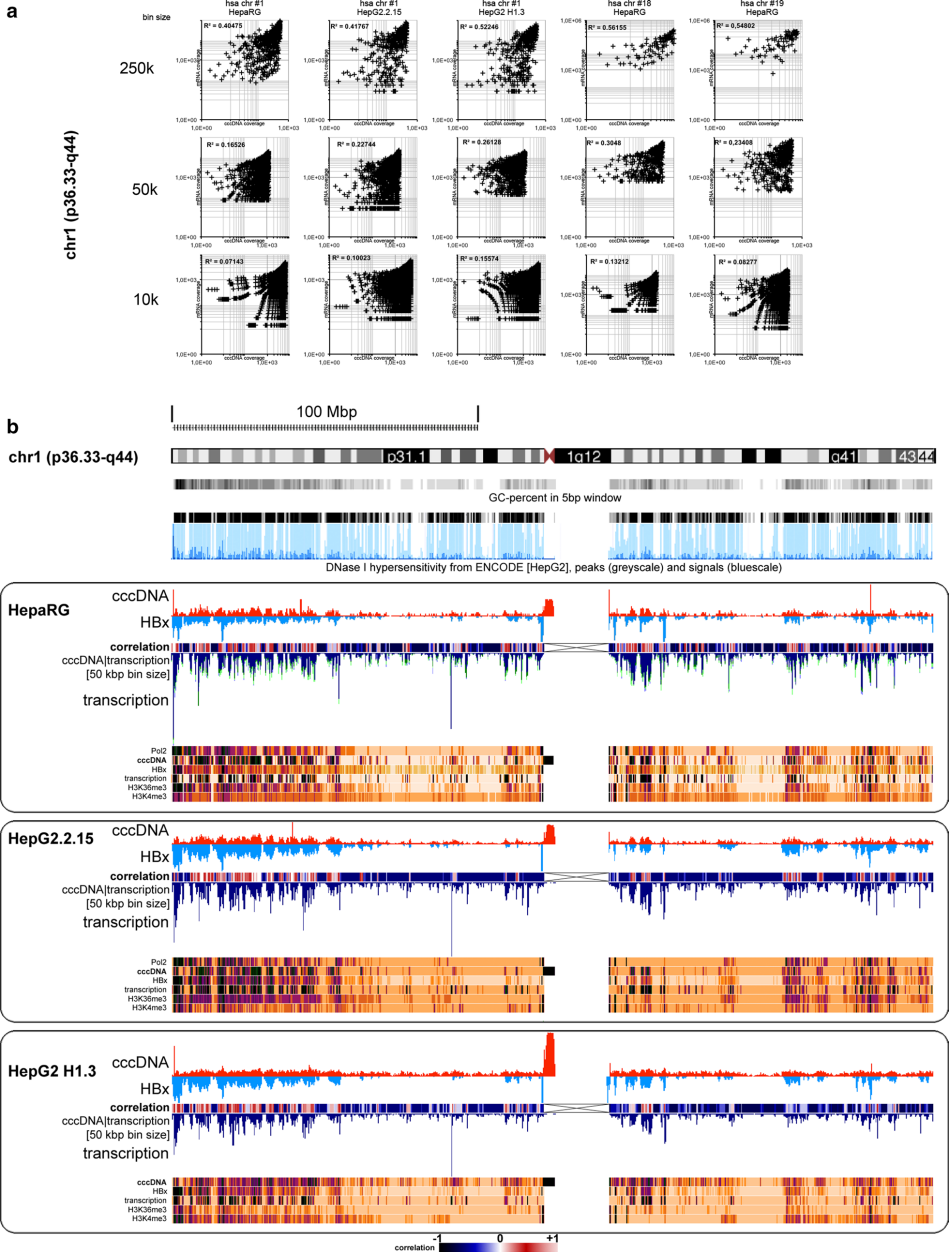
Correlative analyses of HBV cccDNA-hepatocyte genome association and several chromatin structure markers on chromosome 1 in HepaRG, HepG2.2.15 and HepG2 H1.3 cells. **a** We performed correlation analyses between sites of HBV cccDNA enrichment with respect to transcribed regions. To assess the resolution of putative correlations, we applied different bin sizes for analyses. The graphs quintessentially illustrate chromosome 1 in three different cell lines (HepaRG, HepG2.2.15, HepG2H1.3) and chromosomes 18 and 19 in HepaRG cells. Importantly, R^2^ indicates best correlation when larger resolution windows of 50 kb or 250 kb were selected. **b** At the top a human chromosome 1 ideogram is outlined as a reference, including GC content and DNase I hypersensitivity (both taken from the UCSC genome browser). DNase I hypersensitivity peaks are presented in grayscale and signals in blue scale. Below, genome-wide data from 4C and ChIP-seq mapping of HBx and cccDNA are presented for chromosome 1 in three different human hepatocyte cell lines (HepaRG, HepG2.2.15 and HepG2 H1.3 cells), each in a separate box. For each cell line (from top to bottom) this figure shows 4C-derived cccDNA enrichment, HBx enrichment (displayed as a negative signal for the sake of readability), the degree of correlation of cccDNA and genomic mRNA transcription and cccDNA transcription (displayed as a negative signal for the sake of readability). For HepaRG the gene transcription track is an overlay of 4 different time points from day 0 of infection to day 12 post-infection. Below indicated are CHIP-seq derived enrichments of cccDNA, HBx, Pol2, H3K36me3 and H3K4me3. The color-coding reference of the degrees of correlation and coverage are indicated in the legend at the bottom of the figure. This figure reveals correlative mapping using a 50 kb bin size for analyses of HBx and cccDNA with respect to the GC content track and DNase I hypersensitivity (top), gene transcription, Pol2, H3K36me3 and H3K4me3 (see also: https://genome.ucsc.edu/cgi-bin/hgTracks?hgS_doOtherUser=submit&hgS_otherUserName=janzop&hgS_otherUserSessionName=hsa_all_cccDNA_HBx_mRNA). *Further examples of human chromosomes 18 (gene poor) and 19 (gene rich) are shown in* Additional file 1: Figure S5

4C-seq and mRNA-seq analyses yielded comparable patterns of correlative HBV DNA and mRNA enrichment on human chromosomes in all three cell lines, where we observed a robust tendency of positive correlation of cccDNA and mRNA, when larger bin sizes of 50 kb or 250 kb were selected (Fig. 3a). Figure 3b demonstrates the HBV DNA coverage emphasizing on cccDNA on chromosome 1—the largest human chromosome—in HepaRG cells, HepG2.2.15 and HepG2 H1.3. For further background information, Additional file 1: Figure S5 shows the HBV DNA coverage of chromosome 18—the human autosome with the lowest gene density—and chromosome 19—the human autosome with the highest gene density—in HepaRG cells. To interpret these results in the context of chromatin structure, we compared the cccDNA coverage patterns with transcribed mRNA patterns, which we obtained from RNA-seq experiments on cells from the same experiments (since the level of mRNA synthesis is the result of the associated epigenotype).

All patterns of cccDNA coverage on human chromosome maps were strikingly reminiscent of patterns obtained by mRNA-seq read mapping to the human genome. A high level of cccDNA read enrichment was reflecting regions of elevated gene expression activity. Centromers revealed a consistently observed exception: here, a high coverage was observed for HBV DNA, but not for mRNA reads. Further evaluation revealed those reads to consist of highly repetitive sequences abundant in centromeric regions, most likely rendering this observation a methodological artifact. We therefore did not dedicate specific attention to these regions. To analyze the correlation between the read coverages of cccDNA and mRNA, we used the bamCoverage function in deepTools, Galaxy, to divide the genome into bins of specified sizes [44, 45]. Next, we applied bigwigCompare to detect regions of overlapping enrichment within the specified bin size. The best correlation was achieved, when the bin size was adjusted to values (e.g., 50 kb bin size), which could correspond to chromatin fiber structures or the lower size limits of higher-order TAD structures, respectively. In contrast, with lower bin sizes the observed overlap between cccDNA and mRNA coverages was weaker, indicating that the association of cccDNA with transcribed genomic regions did not take place on lower levels of nuclear architecture, i.e., on an oligonucleosomal level or via its association with specific genes. Notably, no cccDNA above-threshold enrichment was observed for chromosomal regions that do not synthesize considerable amounts of mRNA.

To test whether the above-described results for NLS-HBx-RFP hold true for native HBx in HBV-positive human hepatocytes, we compared HBx nuclear localization with the cccDNA. Specifically, we used the mouse monoclonal anti-HBx antibody (clone 3F6-G10) [39] for ChIP-seq of genomic DNA associated with chromatin fractions, which were isolated from purified hepatocyte nuclei. Strikingly, HBx coverage patterns on human chromosomes most closely corresponded to cccDNA patterns. Centromeric sequences were the only exception, as they were not enriched in the purified HBx-containing chromatin immunocomplexes. These observations strongly suggest the probability of the presence of HBx to overlap with that of HBV cccDNA and, moreover, confirmed that native HBx was associated with hepatocyte chromatin. Similarly, we performed ChIP-seq experiments using Pol2 antibodies and selected open chromatin markers (H3K36me3 and H3K4me3) to investigate whether the HBV episomes inhabited an open chromatin environment. This would be expected when HBV episomes were associated with sites of active transcription. All patterns exhibited large overlaps and were reminiscent of previously observed patterns for NLS-HBx-RFP in MMH-D3 cells: While the cccDNA-associated reads mostly covered host genomic regions of active gene transcription (mRNA-seq reads enrichment), there was also overlapping enrichment of Pol2 as well as of H3K36me3 and H3K4me3 (Fig. 3b). This correlation was observed on a genome-wide scale. For the sake of readability exemplary data are shown on human chromosome 1 only for all three HBV-positive cell lines. Furthermore, coverage data from infected human HepaRG cells for cccDNA, HBx and mRNA reads on the gene-poorest human autosome (chromosome 18) and the gene-richest chromosome 19 are displayed as Additional file 1: Figure S5.

### Episomal HBV DNA co-precipitates with native HBx in HepaRG cells, and episomes adopt a chromatin structure reminiscent of their host nuclear habitats

**Fig. 4:**
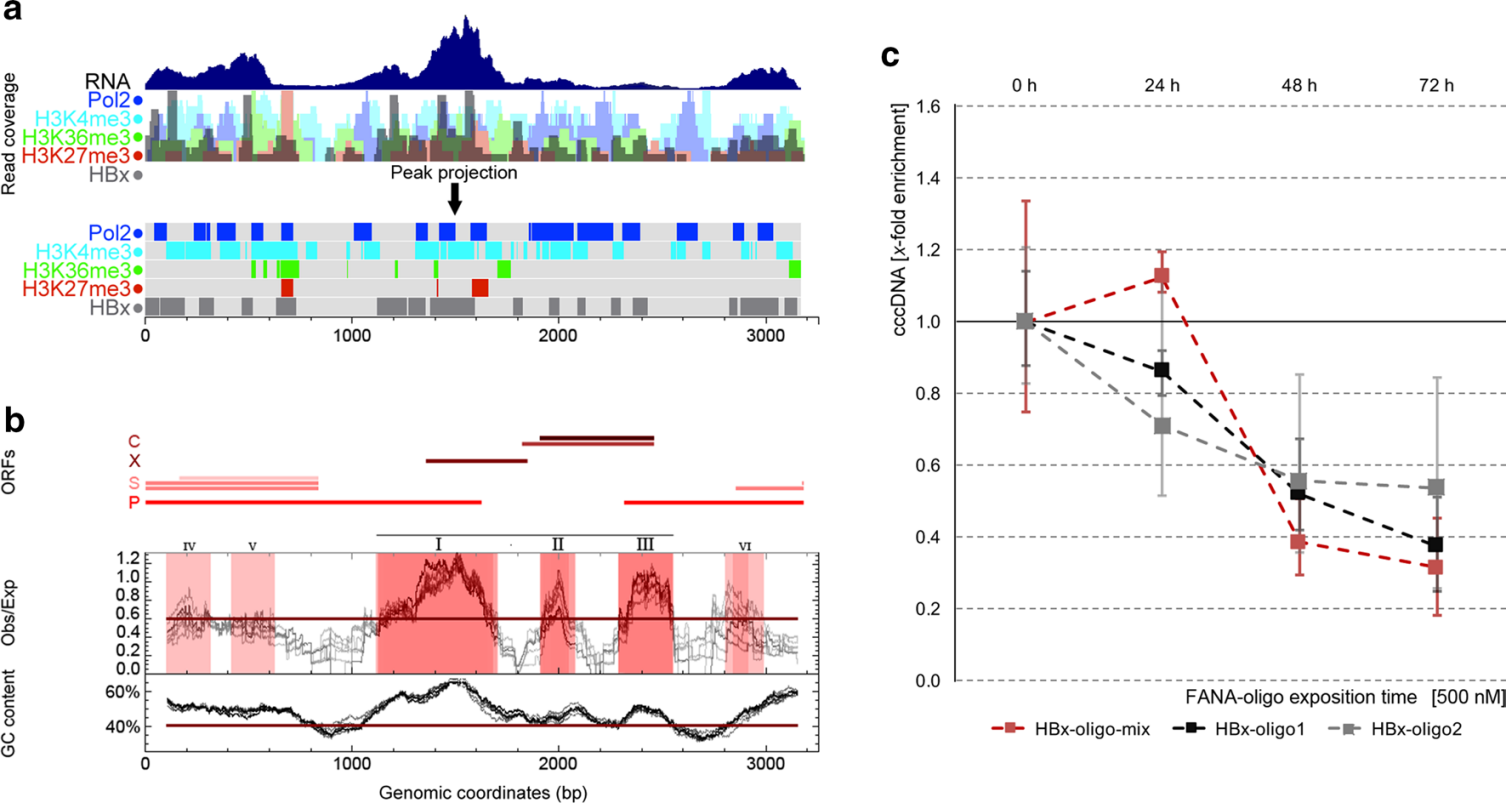
Several euchromatin markers are enriched at HBV cccDNA, and suppression of HBx-mRNA expression via FANA antisense oligonucleotides leads to a reduction in episomal HBV DNA in HepG2.2.15 cells. **a** This figure outlines (from top to bottom) RNA-seq data for cccDNA mRNA transcription (consensus data from HepaRG, HepG2.2.15 and HepG2 H1.3), as well as ChIP-seq derived enrichment of Pol2 (dark blue tracks), H3K4me3 (cyan), H3K36me3 (green), H3K27me3 (red) and HBx (gray). Enrichment peaks are indicated at the top, and read coverage is presented below. Results from RNA-seq suggest that remarkable amounts of mRNA are synthesized from ORF S and ORF X. ChIP-seq reveals that in these cells ‘active’ transcription markers, such as Pol2 and H3K4me3, are enriched at HBV cccDNA. The highest amount of H3K36me3 seemed to be present at the 3′-ends of ORF S and ORF X, whereas comparably low amounts of H3K27me3 were associated with cccDNA. **b** This diagram presents the localization of ORFs, CpG islands (major CpG islands: I–III; minor CpG islands not detected in all genotypes: IV–VI) and the GC content (adapted from Hensel et al. [66]). **c** This figure outlines a time course of episomal HBV DNA quantification by PCR in HepG2.2.15 cells under the influence of FANA oligonucleotide treatment targeting HBx-mRNA. Specifically, two different oligos (HBx-oligo1/HBx-oligo2) or an oligo mixture (HBx-oligo-mix) was used to suppress HBx-mRNA. For normalization, we performed similar experiments where a scrambled non-sense FANA oligonucleotide was used. This figure demonstrates the increasing attenuation of HBV DNA over time under the influence of FANA oligonucleotide anti-HBx treatment

The previous observations shed light on the specific nuclear compartments, wherein the HBV episomes reside persistently. Subsequently, we focused on HBV epigenome analyses to discover, whether this nuclear invader mimics the host chromatin signature on a small nucleosomal level. We utilized HBV-positive HepaRG cells to analyze the correlative mRNA read coverage of the HBV episome by mRNA-seq as well as its association with native HBx, Pol2, H3K4me3, H3K36me3 and H3K27me3 by ChIP-seq (Fig. 4a). We investigated the genomic association of these markers in correlation to specific features associated with the consensus genome of HBV genotypes A–H, i.e., the positions of ORFs P, S, X, C, the three major and three minor CpG islands and the overall GC content (Fig. 4b). These data confirmed the cccDNA in infected HepaRG to be actively transcribed, whereby ORFs S and X appeared to be pronounced at the early stage of infection under investigation (i.e., approx. 12 days post-infection). The HBV cccDNA was furthermore densely associated with HBx, Pol2 and open chromatin markers such as H3K4me3 and H3K36me3. Moreover, we found CGI1 and CGI3 on the cccDNA to occur most probably in a hypomethylated state in infected liver cells from human patients, when compared with circulating HBV DNA (Additional file 1: Figure S6). Interestingly, adjacent to CGI1 are two HBV enhancers, Enh1 and Enh2 [8]. It can be speculated that the influence of these enhancers in an activated state could exceed the involvement of HBV genes. In the nuclear environment—at a TAD-resolution—those enhancers could also trigger proximal host genes in a way reminiscent of enhancer adoption as previously described [54, 55]. Our findings regarding the chromatin signature of the cccDNA are in agreement with previous findings, indicating the cccDNA to be associated with acetylated histones H3 and H3, histone acetyltransferases and several transcription factors characteristic for actively transcribed chromatin [13, 52]. In contrast, we observed that the repressive marker H3K27me3 was only marginally associated with the cccDNA.

### Knock down of native HBx expression weakens the episomal stability of HBV cccDNA in cycling HepG2.2.15 cells

While there appears to be a large overlap between the chromatin signatures of the nuclear habitats of both NLS-HBx-RFP and HBV cccDNA, it remains unknown whether both interact within their niches. To explore whether the episomal stability of the cccDNA changes when the expression of HBx-mRNA becomes silenced, we utilized FANA antisense oligonucleotides for knock down experiments in cycling HepG2.2.15 cells (Fig. 4c). For comparison, we made use of two different anti-HBx-mRNA oligonucleotides (HBx-oligo1/HBx-oligo2) and a mixture of both (HBx-oligo-mix). In all treatment conditions, the concentration of HBV episomes dropped markedly after at least 48 h post treatment and thereafter, indicating that their episomal stability might depend on the expression of HBx.

Taken together, we discovered that a reporter NLS-HBx-RFP construct (in murine hepatocytes) and native HBx as well as HBV episomes (in HBV-positive human hepatocytes) exhibited specific patterns of genome-wide enrichment. Particularly, enrichment was associated with sites of active mRNA synthesis and Pol2 enrichment as well as with several open chromatin markers. These interactions probably took place on domains associated with a higher-order nuclear topology, but not necessarily on a lower level of nuclear organization (i.e., oligonucleosomal level or association with specific genes). Domain depletion experiments in NLS-HBx-RFP demonstrated that HBx-binding to HBV episomes depended on its C-terminus and that knock down of HBx-mRNA in HepG2.2.15 cells lead to a decrease in episomal HBV DNA enrichment, allowing us to speculate that HBx might be functionally involved in the persistent nuclear localization of HBV episomes.

## Discussion

HBx is necessary for the initiation and maintenance of viral replication in cell culture [11] and in human hepatocyte chimeric mice [56], as well as for hepatocarcinogenesis [57]. HBx was previously demonstrated to interact with proteins involved in important nuclear functions. These include DNA damage-binding protein 1 (DBB1) [58], CREB binding protein (CBP)/p300 (a histone acetyltransferase (HAT)), and H4, H4ac, H3ac, PCAF. Moreover, HBx mutant HBV was found to be differentially associated with HDAC1 [59]. These reported interactions may reflect true interplay, which would correspond well to our observations of the nuclear localization of HBV episomes as described above. The most important difference between our approach and the latter study on HBV epigenomics by Belloni et al. is the comprehensive nuclear perspective underlying the current study. Belloni and co-workers utilized several selected antibodies for pull-down assays to test the association of their specific antigens with the cccDNA. They concluded some of the investigated chromatin-associated proteins become recruited to the cccDNA. Our study, on the other hand, involves the view on the host chromatin signature of the nuclear habitat in which the HBV episomes are embedded upon infection.

Remarkably, this is the first study utilizing circular chromosome confirmation capture (4C) and high-throughput sequencing to assess the intra-nuclear localization of cccDNA and the role of HBx for the HBV episome localization. Moreover, to our knowledge this is the first utilization of 4C technology to investigate the host cellular interaction of any viral genome to date. Based on the findings from the present study, the major determinant for the HBV episome localization appears to be an open chromatin structure that is active in transcription. Moreover, probably only very specific, non-redundant factors (i.e., transcription factors) are involved in the process of subnuclear HBV localization.

**Fig. 5:**
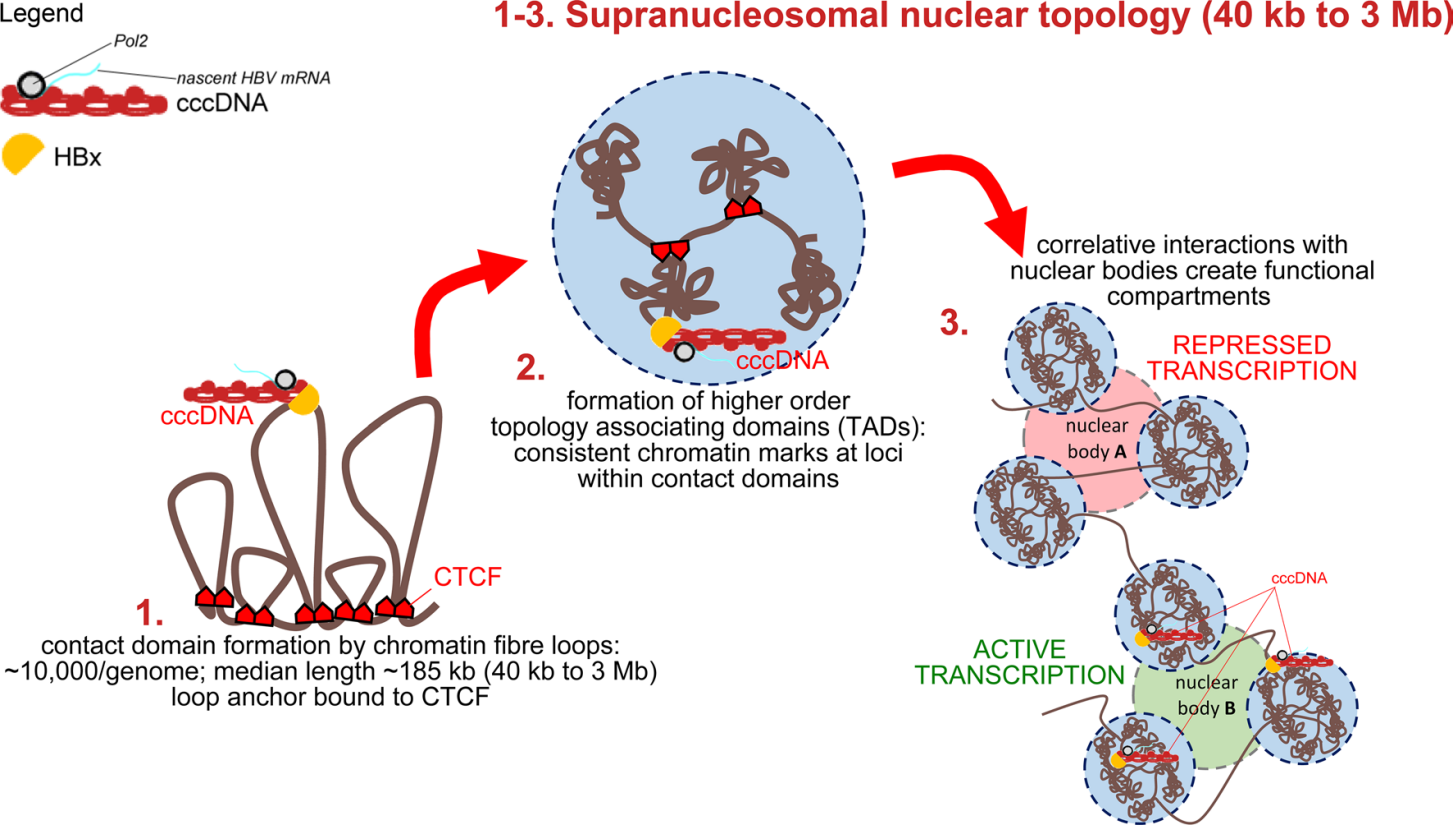
Localization of HBx and HBV episomes in the context of 3D nuclear topology. The graphic illustrates a model for the implementation of activity-associated nuclear HBV localization into current concepts of nuclear higher-order organization. HBx is associated with the HBV episome via C-terminal binding and this complex translocates to sites of active gene expression, which were previously proposed to be organized as focal ‘transcription factories’—functional assemblies of factors and templates involved in transcription—residing in the interchromatin compartment. Actively transcribed chromatin extrudes from the surface of supranucleosomal chromatin structures (domain units) into an adjacent perichromatin compartment to come in contact with transcription factories and other processing machineries [18–20, 24]

Shifting the perspective to the host cell nuclear landscape-centered view and the localization of HBV genomes therein, our findings can be implemented into current views on functional nuclear higher-order organization. Accordingly, HBV episomes may become actively and continuously localized to transcription sites through association and assimilation to actively transcribed chromatin structures. In this way HBV may exploit functional nuclear organization to facilitate long-term persistence (Fig. 5). Possibly, HBV episomes become concomitantly co-regulated with several host genes, which reside on the same subnuclear structure unit (i.e., TADs), or even influence their regulation. This functional nuclear localization pattern might be of significance for the stable long-term episomal establishment of HBV cccDNA in infected hepatocyte nuclei and, ultimately, for HBV-driven hepatocarcinogenesis. This perspective allows the assumption that HBV cccDNA bound by HBx preferentially associates with sites of active gene transcription. Our results leave open whether the association of the cccDNA with specific chromatin proteins and transcription factors is due to direct recruitment to the cccDNA or, alternatively, due to the proximity of HBV episomes to an open chromatin environment and its interactions with functional nuclear bodies, such as transcription factories. Importantly, our data do not directly show that HBx is bringing the cccDNA genomes to these active chromatin sites. Therefore, HBx cannot be concluded to be the direct causal driving force for cccDNA localization in active chromatin compartments. The discovery of the exact mechanism of HBx-induced cccDNA persistence goes beyond the scope of this study and should therefore be addressed by future investigations.

The works of Peter Cook’s laboratory suggest active transcription sites not to be randomly and diffusely distributed within the nuclear interior, but rather organized as ‘transcription factories,’ i.e., foci-like clusters of Pol2, transcribed chromatin, nascent RNA and other activities. Additionally, these transcription factories could be selectively enriched with transcription factors. They probably reside within or in the proximity of the perichromatin region, encompassing constrained chromatin extruding for transcription from dense chromatin domain clusters. Accordingly, and in agreement with our hypothesis, the activity of a given gene is in part regulated through its intra-nuclear position. In that, genome organization is a dynamic process, and molecular structure and function can determine one another [60]. One explanation for the organization of transcriptional activity into factories is the possibility of effective and cumulative use of cellular resources for this nuclear process. Similarly, economization of cccDNA expression through adaptation to the host nuclear organization could enable HBV replication from as little as a few genome copies per cell. Eventually, this might be relevant for its long-lasting low cytotoxicity. HBV cccDNA may necessarily localize to these functional domains to be transcribed and episomally maintained throughout many cell cycles. This is in line with an observation on episomes designed for gene therapy. Specifically, the transfected episomes could only be stably maintained, when they became localized to open chromatin compartments, whereas heterochromatin localization was associated with integration events or loss [48]. This spatiotemporal dependency on episomal stability would also provide an explanation for another yet unexplained phenomenon. While our chip-seq analyses give rise to the above-described potential explanation as a mechanism involving 3D functional nuclear architecture, this work does not definitively prove this theory. On the other hand, the high 50 kbp correlation could be the result of a preference of the virus for open chromatin due to improved accessibility (i.e., to minimize steric hindrance). Further studies are needed to ultimately answer this currently open question.

In a well-conducted study, van Breugel and co-workers demonstrated that HBx—when bound to UV-damaged DNA binding protein 1 (DDB1), a protein which is implicated in DNA repair and transcriptional control of UV-damage induced genes—selectively stimulates the expression of transiently transfected episomal reporter plasmids, regardless of the presence or absence of regulatory sequences. Intriguingly, none of these reporters was stimulated by HBx when integrated into the host genome. This suggests the existence of a yet unexplained selective transactivation mechanism, where HBx specifically targets episomal DNA templates, but not integrated DNA of the same sequence [61]. A valid explanation for this selective transactivation pattern, which apparently does not affect integrated sequences, would be its predominance in inactive chromatin compartments that have no access to active transcription compartments. Possibly, chromosomally integrated HBV DNA may occur preferentially in a more rigid, topologically constrained chromatin structure and could therefore less likely be translocated to transcription factories, i.e., by HBx. Accordingly, the higher-order chromosome topology would impede the motility of integrated HBV genomes. A similar phenomenon has been described for the intra-nuclear behavior of another host cellular genome invader, the human immunodeficiency virus (HIV). The maintenance of integrated HIV genome latency is known to involve transcriptional gene silencing through less accessible chromatin structures, which involves DNA CpG methylation and the activities of histone deacetylases as well as the histone methyltransferase Suv39H1 [62, 63]. On the other hand, drugs antagonizing repressive chromatin structures lead to HIV reactivation [62, 63]. In contrast, and in support of our data it seems convincing that the specific enrichment of HBV episomes at regions of active gene transcription would be favored by its genome organization as small episomes of approx. 3.2 kb DNA in size only, which are naturally not likely subject to motility constrains. Interestingly, while the overall correlation is undeniable, the degree of HBx and cccDNA enrichment correlation varies between specific cell lines and genomic regions. This could be explained by the disequilibrium of intra-nuclear HBx and cccDNA, the latter being much less abundant [64]. Ultimately, a definitive explanation will be subject to further investigations.

## Conclusions

Our observations provoke the question whether the HBV episome chromatin structure could be cause or consequence of its nuclear localization. Importantly, HBx and cccDNA are favorably associated with actively transcribed chromatin. However, while the concept of HBx as an active recruiter for cccDNA to transcribed chromatin regions is a tempting model, this has yet to be addressed by further studies. Foregoing works have shown HBx—in structural association with the HBV episome—to play a crucial role for the establishment of the enduring HBV persistence. In agreement with this, HBV episomal stability becomes weakened when the expression of native HBx-mRNA is silenced by FANA antisense oligonucleotides in cycling HepG2.2.15 cells.

Nevertheless, (early in the HBV life cycle) after nuclear entry, the establishment of HBV cccDNA episomality is based on the conversion of the partially single-stranded HBV RC DNA to the double-stranded cccDNA at a time, when HBx is virtually not yet available. Since RC DNA/cccDNA conversion is linked to DNA repair, it seems reasonable to speculate that DNA repair mechanisms might be exploited by HBV to infiltrate a favorable chromatin environment. It is well known that in response to DNA damage chromatin undergoes a rapid local de-condensation, and that repair kinetics act much faster in an euchromatin context when compared with heterochromatin. Concomitantly, histone PTMs facilitating chromatin fiber accessibility occur, and this is associated with an elevated turnover of nucleosomes. Some structural changes of chromatin are known to spread up to 1 Mb from the site of DNA damage [65]. Hence, the topological chromatin changes associated with DNA repair during RC DNA/cccDNA conversion could provide an entry point for the establishment of HBV episome persistence. Specifically, the peculiar structure of the RC DNA could serve as bait to attract the DNA damaging sensing machinery, entailing events, which eventually lead to the assimilation of HBV episome into the host cell’s active chromatin landscape. This concept provides an exciting hypothetical background for future research directions.

## Additional files

**Additional file 1.** This file contains a collection of additional illustrations (Figures S1–S6) and one table (TableS1).

**Additional file 2.** RNA-seq derived gene expression results of HBV-negative and HBV-positive HepaRG cells as well as HepG2.2.15 and HepG2H1.3 cell lines (Excel spreadsheet).

## Abbreviations

4C: circular chromosome conformation capture
cccDNA: covalently closed circular DNA
FANA: 2′-deoxy-2′-fluoro-beta-D-arabinonucleic acid
HBsAg: hepatitis B surface antigen
HBV: hepatitis B virus
HBx: HBV X protein
HCC: hepatocellular carcinoma
NES: nuclear export signal
NLS: nuclear localization signal
NTCP: Na^+^-taurocholate cotransporting polypeptide
pgRNA: pregenomic RNA
RC DNA: relaxed circular DNA
TAD: topologically-associating domain
