# Supplementary material for: Episomal HBV persistence within transcribed host nuclear chromatin compartments involves HBx
Source: Epigenetics Chromatin. 2018 Jun 22;11:34. doi: 10.1186/s13072-018-0204-2 (PMC6015472; doi:10.1186/s13072-018-0204-2)
Supplement: Supplementary file 1 — Additional file 1. This file contains a collection of additional illustrations (Figures S1–S6) and one table (TableS1). [file 13072_2018_204_MOESM1_ESM.pdf]

Supplementary Material:

Episomal HBV persistence within transcribed host nuclear chromatin compartments involves HBx

Kai O. Hensel[1], Franziska Cantner[1], Felix Bangert[1], Stefan Wirth[1] and Jan Postberg[1,2]

[1]Department of Pediatrics, HELIOS University Hospital Wuppertal, Centre for Clinical & Translational Research (CCTR), Faculty of Health, Centre for Biomedical Education & Research (ZBAF), Witten/Herdecke University, Heusnerstr. 40, 42283 Wuppertal, Germany

[2]Clinical Molecular Genetics and Epigenetics, Faculty of Health, School of Medicine, Centre for Biomedical Education & Research (ZBAF), Witten/Herdecke University, Alfred-Herrhausen-Str. 50, 58448 Witten, Germany

e-mail addresses: kai.hensel@uni-wh.de (Kai O. Hensel); franziska.cantner@uni-wh.de (Franziska Cantner); fba@felixnelson.de (Felix Bangert); stefan.wirth@uni-wh.de (Stefan Wirth); jan.postberg@uni-wh.de (Jan Postberg)

Correspondence:

Prof. Dr. rer. nat. Jan Postberg

Clinical Molecular Genetics and Epigenetics, Faculty of Health, School of Medicine, Centre for Biomedical Education & Research (ZBAF), Witten/Herdecke University, Alfred_Herrhausen_Str. 50, 58448 Witten, Germany

Phone: (+49)202-896-2540

FAX: (+49)202-896-2546

Email: jan.postberg@uni-wh.de

Supplemental data legends

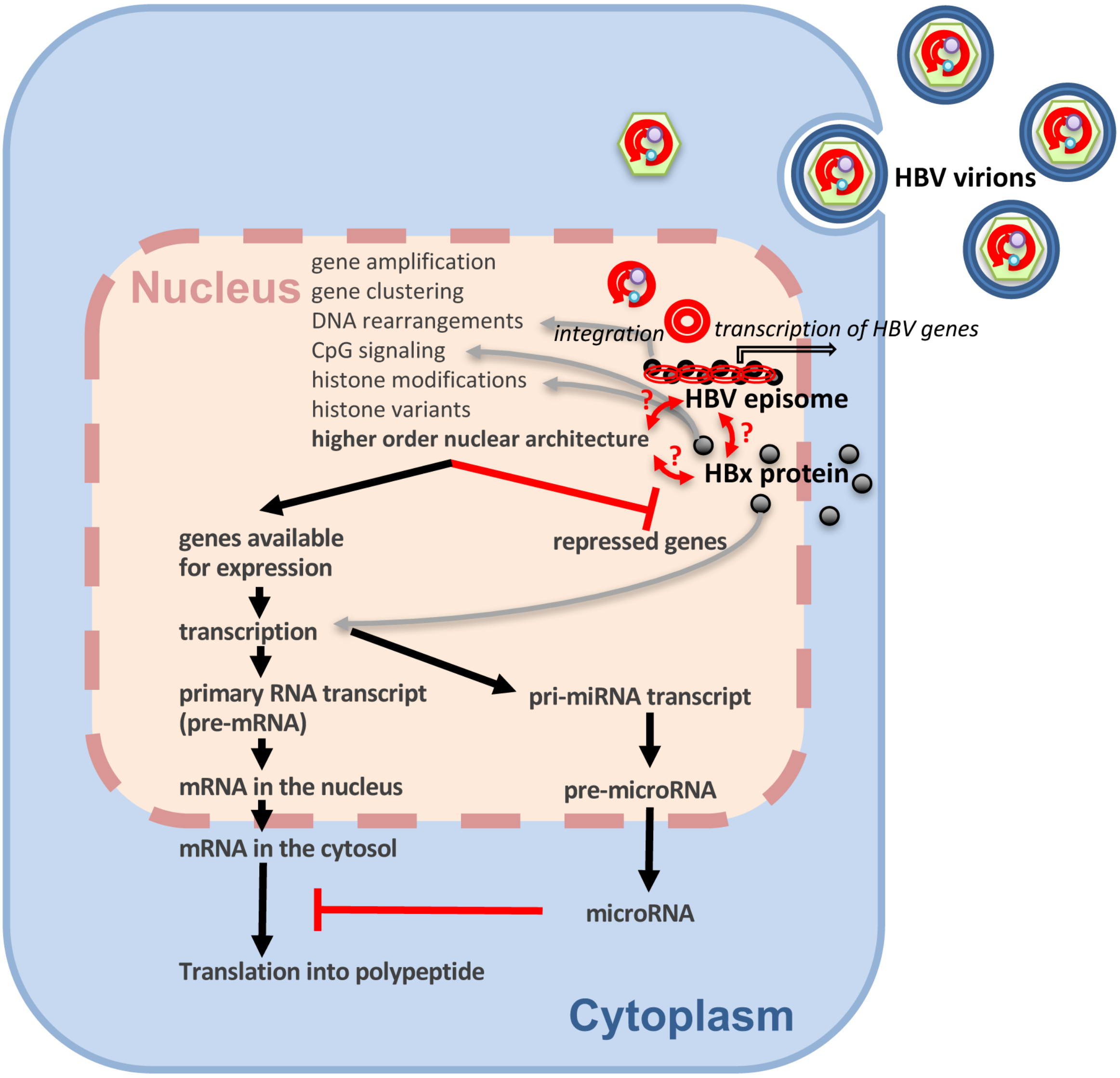

Supplemental data (Figure S1). Illustration showing the influence of HBV on host gene expression.

In the course of HCC development gene expression in hepatocyte nuclei can be influenced through HBV on both the genome and the epigenome levels. Influence on the genome level involves integration of HBV DNA into the host genome. Further, HBx is thought to modulate the expression of host genes via regulation of CpG signaling and histone modification patterns. To date, the interactions of the cccDNA and HBx with the 3D host higher order nuclear architecture remain unexplored.

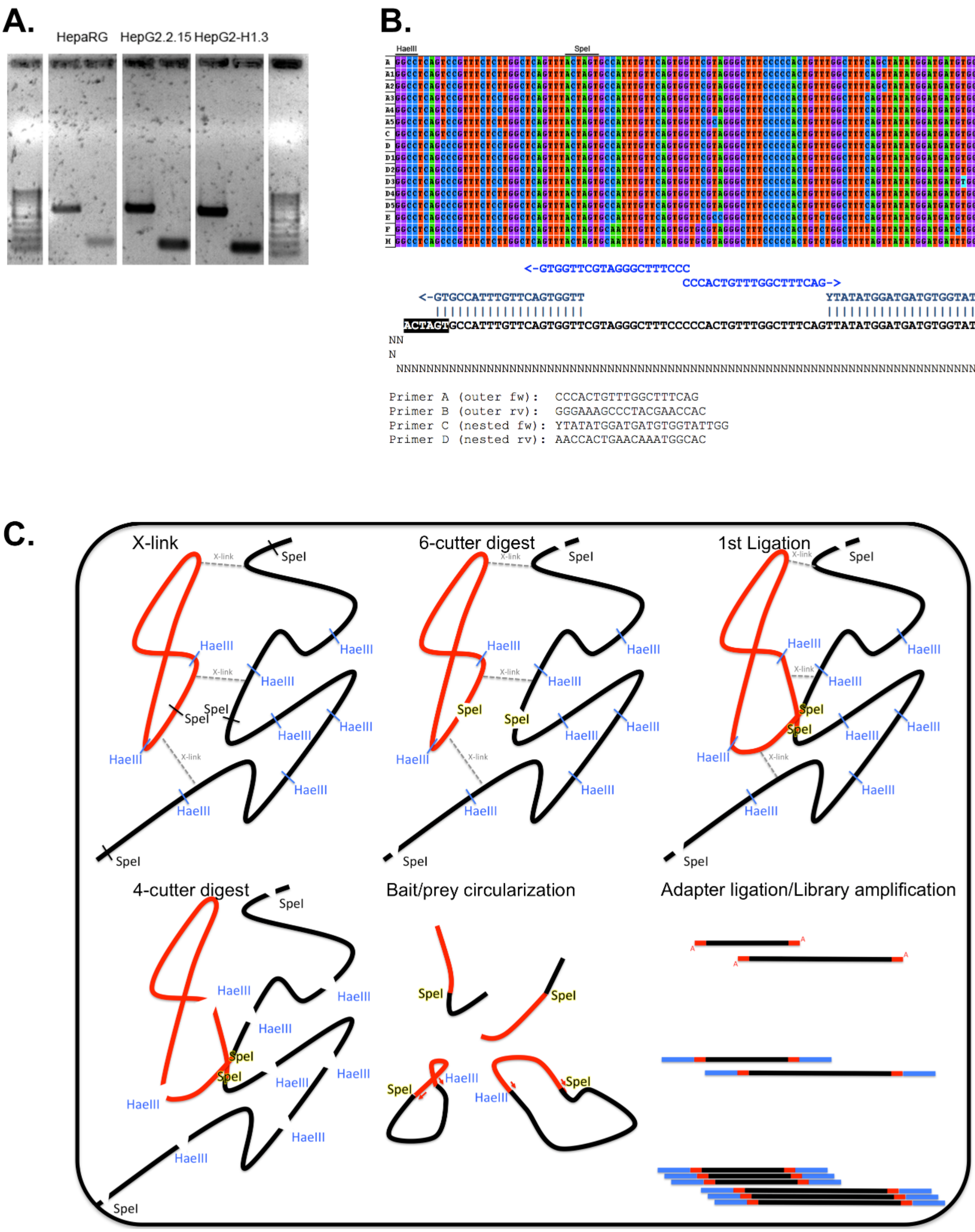

Supplemental data (Figure S2). Illustration of selected methods.

A. cccDNA amplicons from several cell lines were separated on agarose gels to monitor the replication of HBV. These amplicons were further used for Sanger sequencing to verify the HBV-D3 genotype.

B. Alignment of an HBV genomic region-of-interest containing a conserved SpeI restriction site flanked by 2 HaeIII restriction sites. PCR strategy to amplify host genomic DNA from cccDNA-host genome hybrids is depicted below. C. Principle and work flow of the 4C method.

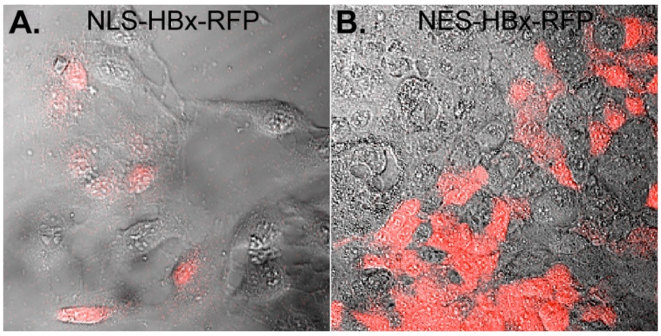

Supplemental data (Figure S3). Fluorescence microscopy of NLS- or NES-HBx-RFP-transfected hepatocytes *in vitro*. Verification of RFP-fusion protein expression in transfected cells demonstrating nuclear (A) or extranuclear (B) localization.

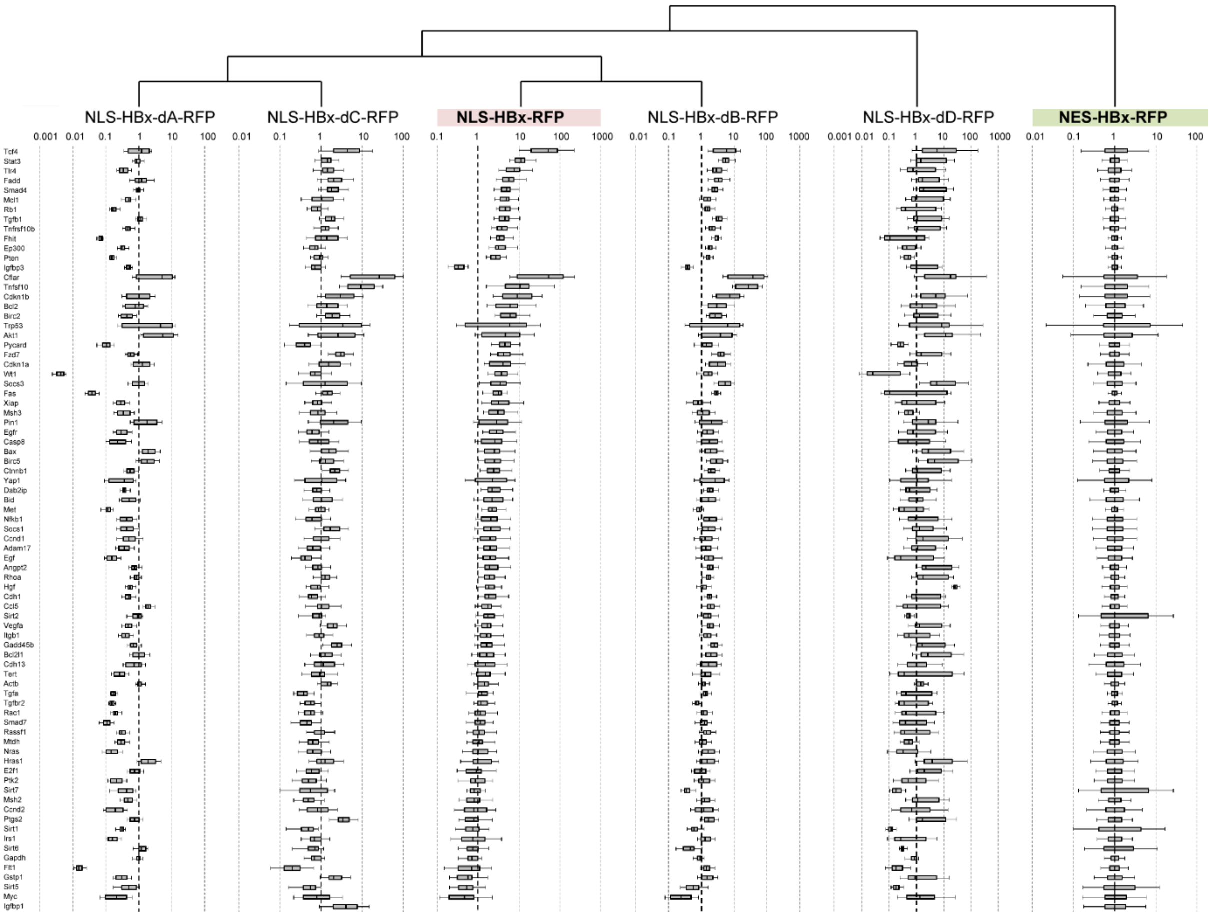

Supplemental data (Figure S4). qPCR-derived gene expression patterns of murine hepatocytes transfected with several different HBx constructs and comparative analyses of the consequences of directed HBx-domain depletion.

MMH-D3 hepatocytes were transfected with different NLS-HBx-RFP plasmid constructs (wild type HBx and four different partial deletion constructs: ΔA-ΔD; compare to Figure 2A). Relative enrichment of mRNAs relevant for the development of HCC are outlined subjected to unsupervised hierarchical cluster analysis assembly according to differences in gene expression patterns.

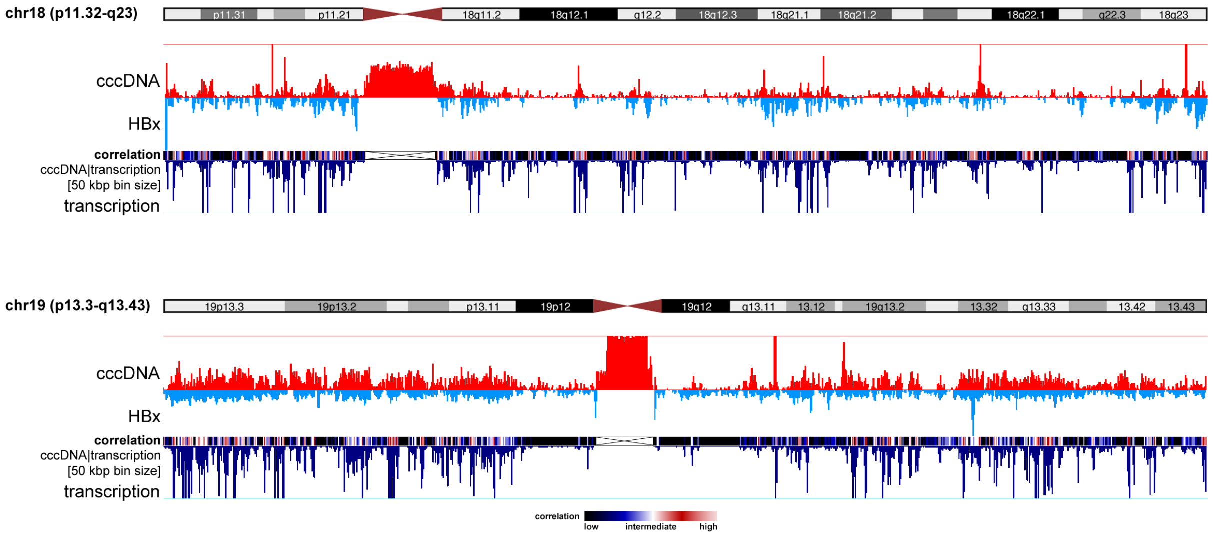

Supplemental data (Figure S5). Correlative analyses of HBV cccDNA-HepaRG genome association and actively transcribed genes.

The gene-poor human chromosome 18 and gene-rich chromosome 19 are shown as further examples to complement Figure 3.

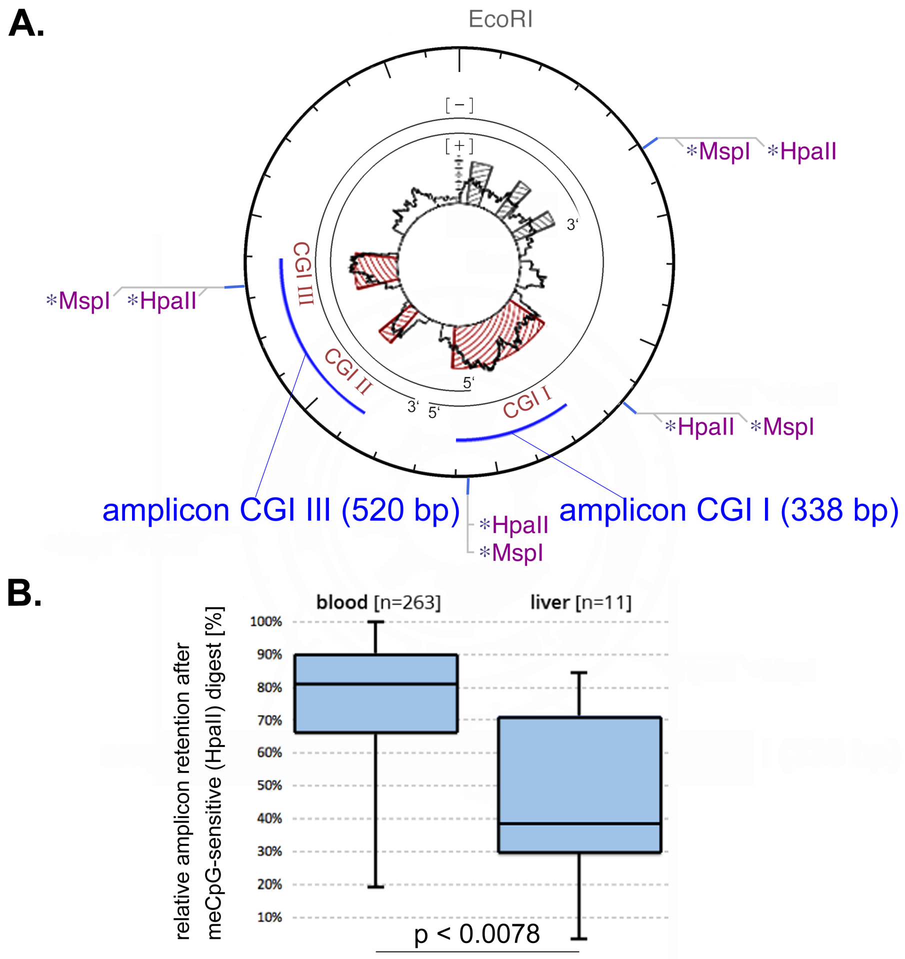

Supplemental data (Figure S6). Comparing DNA methylation between circulating HBV DNA and cccDNA from patients' liver cells.

A. The HBV genome map illustrates the position of 3 major CpG islands (CGIs, red boxes covering the GC content graphs – grey boxes illustrate the position of putative CGIs not conserved in all HBV genotypes [1]) and HpaII/MspI restriction sites with respect to important RC DNA features. Two amplicons covering HpaII/MspI restriction sites within CGI1 or CGI2, respectively, were used for qPCR analyses. Primers were: HBV_CGI1fw: 5'-ccgatccatactgcggaactcct-3' with HBV_CGI1rv: 5'-agaggtgaagcgaagtgcacac-3' or HBV_CGI3fw: 5'-agctgtgccttgggtggcttt-3' with HBV_CGI3rv: 5'-ctgcgaggcgagggagttc-3', repectively. DNA methylation was determined using a combination of DNA-methylation sensitive restriction endonuclease digest (HpaII/MspI) and qPCR. 2 amplicons were analyzed and compared. We prioritized this approach over bisulfite-based approaches due to the limited amount of HBV DNA in many small specimens. Another reason was that the selected approach favors the analysis of cccDNA over RC DNA due to the substrate-specificity of restriction endonucleases on double

stranded DNA (at least one HpaII/MspI-site of interest in CGI1 corresponds to the ssDNA region of RC DNA). We found that the combinatorial use of DNA methylation-sensitive endonuclease digest and qPCR allowed us a robust detection of HBV DNA retention after HpaII digest, when in parallel compared with MspI digest on even very low amounts of HBV DNA isolated from patient's sera or liver biopsies. B. It turned out that after HpaII digests the amount of detected HBV DNA from blood (median: 81.30% [25%ile: 66.36%/75%ile: 90.03%] for n=263 with respect to HBV DNA from the same samples digested with MspI) was significantly (p<0.0078) higher than for HBV DNA from infected human liver (median: 38.59% [25%ile: 29.54%/75%ile: 71.12%] for n=11 with respect to HBV DNA from the same samples digested with MspI). These data suggest that the cccDNA in infected human liver cells occurs in a hypomethylated state, if compared with circulating HBV DNA.

Supplemental data (Table S1). Gene list qPCR panel PAMM-133R 'liver cancer' (Qiagen, Hilden, Germany)

Position	Unigene	Refseq	Symbol	Description
A01	Mm.27681	NM_009615	Adam17	A disintegrin and metallopeptidase domain 17
A02	Mm.6645	NM_009652	Akt1	Thymoma viral proto-oncogene 1
A03	Mm.439874	NM_007426	Angpt2	Angiopoietin 2
A04	Mm.19904	NM_007527	Bax	Bcl2-associated X protein
A05	Mm.257460	NM_009741	Bcl2	B-cell leukemia/lymphoma 2
A06	Mm.238213	NM_009743	Bcl2l1	Bcl2-like 1
A07	Mm.235081	NM_007544	Bid	BH3 interacting domain death agonist
A08	Mm.335659	NM_007465	Birc2	Baculoviral IAP repeat-containing 2
A09	Mm.8552	NM_009689	Birc5	Baculoviral IAP repeat-containing 5
A10	Mm.336851	NM_009812	Casp8	Caspase 8
A11	Mm.284248	NM_013653	Ccl5	Chemokine (C-C motif) ligand 5
A12	Mm.273049	NM_007631	Ccnd1	Cyclin D1
B01	Mm.333406	NM_009829	Ccnd2	Cyclin D2
B02	Mm.35605	NM_009864	Cdh1	Cadherin 1
B03	Mm.334841	NM_019707	Cdh13	Cadherin 13

B04	Mm.195663	NM_007669	Cdkn1a	Cyclin-dependent kinase inhibitor 1A (P21)
B05	Mm.2958	NM_009875	Cdkn1b	Cyclin-dependent kinase inhibitor 1B
B06	Mm.4733	NM_009877	Cdkn2a	Cyclin-dependent kinase inhibitor 2A
B07	Mm.336848	NM_009805	Cflar	CASP8 and FADD-like apoptosis regulator
B08	Mm.291928	NM_007614	Ctnnb1	Catenin (cadherin associated protein), beta 1
B09	Mm.1401	NM_009911	Cxcr4	Chemokine (C-X-C motif) receptor 4
B10	Mm.29629	NM_001001602	Dab2ip	Disabled homolog 2 (Drosophila) interacting protein
B11	Mm.210875	NM_015802	Dlc1	Deleted in liver cancer 1
B12	Mm.18036	NM_007891	E2f1	E2F transcription factor 1
C01	Mm.252481	NM_010113	Egf	Epidermal growth factor
C02	Mm.8534	NM_007912	Egfr	Epidermal growth factor receptor
C03	Mm.258397	NM_177821	Ep300	E1A binding protein p300
C04	Mm.5126	NM_010175	Fadd	Fas (TNFRSF6)-associated via death domain
C05	Mm.1626	NM_007987	Fas	Fas (TNF receptor superfamily member 6)
C06	Mm.397619	NM_010210	Fhit	Fragile histidine triad gene
C07	Mm.389712	NM_010228	Flt1	FMS-like tyrosine kinase 1
C08	Mm.297906	NM_008057	Fzd7	Frizzled homolog 7 (Drosophila)
C09	Mm.1360	NM_008655	Gadd45b	Growth arrest and DNA-damage-inducible 45 beta
C10	Mm.299292	NM_013541	Gstp1	Glutathione S-transferase, pi 1
C11	Mm.267078	NM_010427	Hgf	Hepatocyte growth factor
C12	Mm.254493	NM_020259	Hhip	Hedgehog-interacting protein
D01	Mm.334313	NM_008284	Hras1	Harvey rat sarcoma virus oncogene 1
D02	Mm.3862	NM_010514	Igf2	Insulin-like growth factor 2
D03	Mm.21300	NM_008341	Igfbp1	Insulin-like growth factor binding protein 1
D04	Mm.29254	NM_008343	Igfbp3	Insulin-like growth factor binding protein 3
D05	Mm.4952	NM_010570	Irs1	Insulin receptor substrate 1
D06	Mm.263396	NM_010578	Itgb1	Integrin beta 1 (fibronectin receptor beta)
D07	Mm.285	NM_010612	Kdr	Kinase insert domain protein receptor
D08	Mm.255219	NM_010703	Lef1	Lymphoid enhancer binding factor 1
D09	Mm.1639	NM_008562	Mcl1	Myeloid cell leukemia sequence 1
D10	Mm.86844	NM_008591	Met	Met proto-oncogene
D11	Mm.4619	NM_008628	Msh2	MutS homolog 2 (E. coli)
D12	Mm.343101	NM_010829	Msh3	MutS homolog 3 (E. coli)
E01	Mm.130883	NM_026002	Mtdh	Metadherin
E02	Mm.2444	NM_010849	Myc	Myelocytomatosis oncogene

E03	Mm.256765	NM_008689	Nfkb1	Nuclear factor of kappa light polypeptide gene enhancer in B-cells 1, p105
E04	Mm.400954	NM_010937	Nras	Neuroblastoma ras oncogene
E05	Mm.379474	NM_177906	Opcml	Opioid binding protein/cell adhesion molecule-like
E06	Mm.221403	NM_011058	Pdgfra	Platelet derived growth factor receptor, alpha polypeptide
E07	Mm.7906	NM_023371	Pin1	Protein (peptidyl-prolyl cis/trans isomerase) NIMA-interacting 1
E08	Mm.245395	NM_008960	Pten	Phosphatase and tensin homolog
E09	Mm.292547	NM_011198	Ptgs2	Prostaglandin-endoperoxide synthase 2
E10	Mm.254494	NM_007982	Ptk2	PTK2 protein tyrosine kinase 2
E11	Mm.24163	NM_023258	Pycard	PYD and CARD domain containing
E12	Mm.292510	NM_009007	Rac1	RAS-related C3 botulinum substrate 1
F01	Mm.12091	NM_019713	Rassf1	Ras association (RalGDS/AF-6) domain family member 1
F02	Mm.273862	NM_009029	Rb1	Retinoblastoma 1
F03	Mm.425236	NM_011261	Reln	Reelin
F04	Mm.757	NM_016802	Rhoa	Ras homolog gene family, member A
F05	Mm.378894	NM_019732	Runx3	Runt related transcription factor 3
F06	Mm.19155	NM_009144	Sfrp2	Secreted frizzled-related protein 2
F07	Mm.100399	NM_008540	Smad4	MAD homolog 4 (Drosophila)
F08	Mm.34407	NM_001042660	Smad7	MAD homolog 7 (Drosophila)
F09	Mm.130	NM_009896	Socs1	Suppressor of cytokine signaling 1
F10	Mm.3468	NM_007707	Socs3	Suppressor of cytokine signaling 3
F11	Mm.249934	NM_011486	Stat3	Signal transducer and activator of transcription 3
F12	Mm.4269	NM_013685	Tcf4	Transcription factor 4
G01	Mm.10109	NM_009354	Tert	Telomerase reverse transcriptase
G02	Mm.137222	NM_031199	Tgfa	Transforming growth factor alpha
G03	Mm.248380	NM_011577	Tgfb1	Transforming growth factor, beta 1
G04	Mm.172346	NM_009371	Tgfbr2	Transforming growth factor, beta receptor II
G05	Mm.38049	NM_021297	Tlr4	Toll-like receptor 4
G06	Mm.193430	NM_020275	Tnfrsf10b	Tumor necrosis factor receptor superfamily, member 10b
G07	Mm.1062	NM_009425	Tnfsf10	Tumor necrosis factor (ligand) superfamily, member 10
G08	Mm.222	NM_011640	Trp53	Transformation related protein 53
G09	Mm.282184	NM_009505	Vegfa	Vascular endothelial growth factor A
G10	Mm.389339	NM_144783	Wt1	Wilms tumor 1 homolog
G11	Mm.259879	NM_009688	Xiap	X-linked inhibitor of apoptosis
G12	Mm.221992	NM_009534	Yap1	Yes-associated protein 1
H01	Mm.3317	NM_010368	Gusb	Glucuronidase, beta

H02	Mm.299381	NM_013556	Hprt	Hypoxanthine guanine phosphoribosyl transferase
H03	Mm.2180	NM_008302	Hsp90ab1	Heat shock protein 90 alpha (cytosolic), class B member 1
H04	Mm.343110	NM_008084	Gapdh	Glyceraldehyde-3-phosphate dehydrogenase
H05	Mm.328431	NM_007393	Actb	Actin, beta

Supplemental data (Table S2). Host cellular gene expression in three different cell lines.

RNA-seq derived host cell gene expression including a color legend is provided in a separate file. HepaRG cells were analyzed immediately before HBV infection (t=0) and subsequently at three different time points (t = 1-3). Moreover, the HBV-positive hepatoma cell lines HepG2.2.15 and HepG2H1.3 were analysed. For data sorting 2 main criteria were applied: 1. lines 13-125 represent 113 out of 157 listed liver marker genes (with reference to the Human Protein Atlas [https://www.proteinatlas.org]). It is notable that - in contrast to HepaRG cell - many of these liver markers exhibit decreased levels in HepG2.2.15 and HepG2H1.3 cells or, respectively, are not detectable. **Table S2 is provided as a separate Supplementary Material file (Excel spreadsheet).**

References

1. Hensel KO, Rendon JC, Navas MC, Rots MG, Postberg J: **Virus-host-interplay in hepatitis B virus infection and epigenetic treatment strategies**. *FEBS J* 2017.
